# Synthetic Development of New 3-(4-Arylmethylamino)butyl-5-arylidene-rhodanines under Microwave Irradiation and Their Effects on Tumor Cell Lines and against Protein Kinases

**DOI:** 10.3390/molecules200712412

**Published:** 2015-07-08

**Authors:** Camille Déliko Dago, Christelle N´ta Ambeu, Wacothon-Karime Coulibaly, Yves-Alain Békro, Janat Mamyrbékova, Audrey Defontaine, Blandine Baratte, Stéphane Bach, Sandrine Ruchaud, Rémy Le Guével, Myriam Ravache, Anne Corlu, Jean-Pierre Bazureau

**Affiliations:** 1 Université de Rennes 1, Institut des Sciences Chimiques de Rennes (ISCR), UMR CNRS 6226, Groupe ICMV, Bât. 10A, Campus de Beaulieu, 263 Avenue du Général Leclerc, CS 74205, 35042 Rennes Cedex, France; E-Mails: deliko.dago@univ-rennes1.fr (C.D.D.); christelle.ambeu@univ-rennes1.fr (C.N.A.); 2 Laboratoire de Chimie Bio-Organique et de Substances Naturelles (LCBOSN), Université Nangui Abrogoua, Abidjan 02, BP 802, Cote d'Ivoire; E-Mails: bekro2001@yahoo.fr (Y.-A.B.); kojanova1926@hotmail.fr (J.M.); 3 UFR des Sciences Biologiques, Université de Péléforo Gon Coulibaly, Korhogo, BP 1328, Cote d'Ivoire; E-Mail: wacothon@yahoo.fr; 4 Station Biologique de Roscoff, USR 3151, CNRS-UPMC, Kinase Inhibitory Specialized Screening facility, KISSf, Place George Teissier, BP 74, 29682 Roscoff, France; E-Mails: audrey.defontaine@sb-roscoff.fr (A.D.); blandine.baratte@sb-roscoff.fr (B.B.); stephane.bach@sb-roscoff.fr (S.B.); sandrine.ruchaud@sb-roscoff.fr (S.R.); 5 Université de Rennes 1, ImPACcell, SFR Biosit, Bât. 8, 2 Avenue du Professeur Léon Bernard, CS 34317, 35043 Rennes Cedex, France; E-Mails: remy.leguevel@univ-rennes1.fr (R.L.G.); myriam.ravache@gmail.com (M.R.); anne.corlu@univ-rennes1.fr (A.C.)

**Keywords:** one-pot two-steps, Knoevenagel condensation, 5-arylidene rhodanine, protein kinase, inhibitor, *Ss*CK1, *Hs*CDK5-p25, cell lines, Alzheimer’s disease, cancer

## Abstract

A new route to 3-(4-arylmethylamino)butyl-5-arylidene-2-thioxo-1,3-thiazolidine-4-one **9** was developed in six steps from commercial 1,4-diaminobutane **1** as starting material. The key step of this multi-step synthesis involved a solution phase “one-pot two-steps” approach assisted by microwave dielectric from *N*-(arylmethyl)butane-1,4-diamine hydrochloride **6a**–**f** (as source of the first point diversity) and commercial bis-(carboxymethyl)-trithiocarbonate reagent **7** for construction of the rhodanine platform. This platform was immediately functionalized by Knoevenagel condensation under microwave irradiation with a series of aromatic aldehydes **3** as second point of diversity. These new compounds were prepared in moderate to good yields and the fourteen synthetic products **9a**–**n** have been obtained with a *Z*-geometry about their exocyclic double bond. These new 5-arylidene rhodanines derivatives **9a**–**n** were tested for their kinase inhibitory potencies against four protein kinases: Human cyclin-dependent kinase 5-p25, *Hs*CDK5-p25; porcine Glycogen Synthase Kinase-3, GSK-3α/β; porcine Casein Kinase 1, *Ss*CK1 and human *Hs*Haspin. They have also been evaluated for their *in vitro* inhibition of cell proliferation (HuH7 D12, Caco 2, MDA-MB 231, HCT 116, PC3, NCI-H727, HaCat and fibroblasts). Among of all these compounds, **9j** presented selective micromolar inhibition activity on *Ss*CK1 and **9i** exhibited antitumor activities in the HuH7 D12, MDA-MBD231 cell lines.

## 1. Introduction

During the last decades, the five-membered heterocycle rings (FMHRs) are considered “privileged scaffolds” in medicinal chemistry [1]. Among the five-membered heterocycle rings, the 5-arylidene-2-thioxo-1,3-thiazolidine-4-ones or 5-arylidene rhodanine derivatives represented particularly privileged moieties in drug discovery because they have an inherent tendency for biological activity [2], such as DDX3 inhibitor for HIV replication [3], as potent and selective inhibitors of the “atypical” dual-specificity phosphatase (DSP) family member-JNK-stimulating phosphatase-1 (JSP-1) [4], as pancreatic cholesterol esterase (CEase) inhibitor [5] with IC_50_ values ranging from 1.44 to 85 μM. To discover chemical probes to further understand the function of human DNA polymerase λ in cancer, an inhibitor high-throughput screening (HTS) using SYBR^®^ Green-base assay [6] revealed that three 5-arylidene-2-thioxo-1,3-thiazolidine-4-ones were identified as strong inhibitors. A new series of D-glutamic acid-base *Escherichia coli* MurD inhibitors incorporating the 5-arylidene rhodanine scaffold have been designed, synthesized and evaluated [7]. Substituted 5-arylidene-2-thioxo-1,3-thiazolidine-4-ones were able to inhibit HIV replication in MT-4 cells at low micromolar concentration with an appreciable selectivity index and are good scaffolds for the development of novel HIV-1 integrase inhibitors [8]. Recently, a family of 5-arylalkylidene rhodanine derivatives presented antiviral activity against chikungunya virus (LR2006_OPY1) in Vero cell culture by cytopathic effect CPE reduction assay [9]. For Alzheimer’s disease, the FMHRs derived from 5-arylidene-2-thioxo-1,3-thiazolidine-4-ones have been described for amyloid polypeptide fibril formation [10], regulation of Cathepsin D immuno-reactivity in the senile plaques [11] and inhibition of tau aggregation [12].

Protein kinases represent an important class of enzymes that play an important role in the regulation of various processes. These enzymes catalyze protein-phosphorylation on serine, threonine and tyrosine residues, which are frequently deregulated in human diseases. Only the 518 human kinases have been investigated as potential therapeutic targets [13]. Consequently, the search of protein-kinase inhibitors represented interesting targets in the pharmaceutical industry for new therapeutic agents. Over the past decade, our research group have investigated the chemical development of five-membered heterocycle rings derived from marine alkaloid as low-molecular weight-inhibitors of dual specificity, tyrosine phosphorylation-regulated kinases (DYRKs) and CLKs (cdc2-like kinases) [14,15,16], two families of kinases involved in various diseases including Alzheimer’s disease (AD) [17], and also cancer [18,19,20].

**Figure 1:**
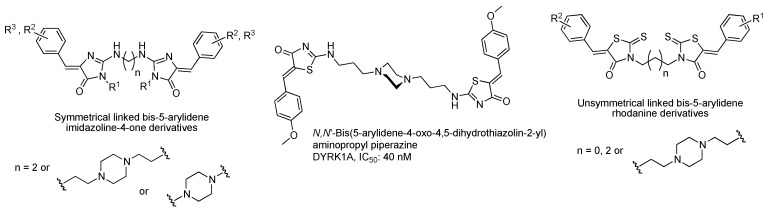
Symmetrical linked bis-5-arylidene imidazoline-4-ones, symmetrical linked bis-5-arylidene-1,3-thiazoline-4-ones and unsymmetrical linked bis-5-arylidene rhodanines derivatives (as inhibitors of the proteine kinase DYRK1A or against cell proliferation on cell tumor lines) identified and developed in our group.

Continuing in the effort to identify new DYRK inhibitors, particularly DYRK1A, we continued to explore successively the synthesis of *N*,*Nʹ*-bis-(5-arylidene-4-oxo-3,5-dihydro-4*H*-imidazol-2-yl)diamines [21], *N*,*Nʹ*-bis-(5-arylidene-4-oxo-4,5-dihydrothiazolidine-2-yl)aminopropylpiperazines [22] and finally unsymmetrical linked bis-5-arylidene-rhodanine derivatives linked in *N*-3 position [23] as potential kinases inhibitors (Figure 1). Among the three series of symmetrical or unsymmetrical *N*,*Nʹ*-diamines bearing various platforms (imidazolidine-4-one or 2-thioxo-1,3-thiazolidine-4-one moieties), only one of these compounds has shown nanomolar inhibition potency (IC_50_ 40 nM) towards DYRK1A. Owing to the fact that none of the unsymmetrical linked bis-5-arylidene rhodanines presented a significant activity against representative tumoral cell lines, we decided to change one of the two heterocyclic platforms by various (arylmethyl)aminobutyl moieties grafted on *N*-3 position of 5-arylidene-rhodanine derivatives in order to obtain significant potential biological activities on tumoral cell lines and effects on some protein kinases (*Hs*CDK5-p25, *Ss*GSK3α/β, *Ss*CK1 and *Hs*Haspin). Herein, we present the building of this 3-(4-arylmethylamino)butyl-5-arylidene-rhodanine library in which the 5-arylidene-2-thioxo-1,3-thiazolidine-4-one moiety was mostly carried out under microwave irradiation [24] and the biological activities of these compounds.

## 2. Results and Discussion

### 2.1. Chemistry

**Scheme 1:**
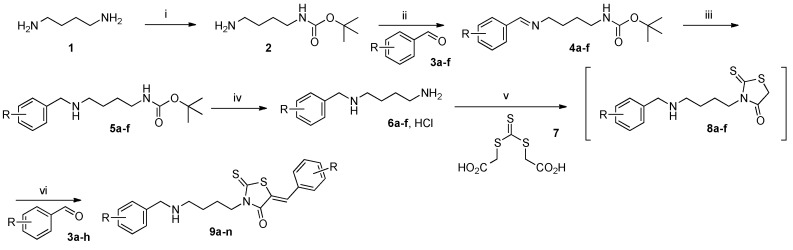
Route used for the preparation of 3-(4-arylphenylamino)butyl-5-arylidene-2-thioxo-1,3-thiazolidine-4-ones 9 via the “one-pot two-steps” reaction under microwave. *Reagents and reaction conditions:* (i) (*t-*BuO_2_C)_2_O, 1,4-dioxane, 25 °C, 24 h. (ii) **3** 1 equiv., molecular sieve 3Å, Et_2_O, 25 °C, 16 h. (iii) NaBH_4_ 5 equiv., MeOH, 50 °C, 24 h. (iv) HCl 6 M, 1,4-dioxane, 25 °C, 4 h. (v) **6** 1 equiv., **7** 1 equiv., Et_3_N 2 or 3 equiv., DME, MWI, 90 °C, 15 or 30 min. (vi) **3** 1 equiv., MWI, 110 °C, 15 or 30 min.

Access to the planned 3-(4-arylphenylamino)butyl-5-arylidene-2-thioxo-1,3-thiazolidine-4-ones **9** is outlined in Scheme 1. For this study, we selected a diamino linker **1** with a butyl chain in order to obtain a good molecular flexibility between the 4-arylmethyl moiety and the 2-thioxo-1,3-thiazolidine-4-one platform. A molecule with the lowest number of carbons for the diamino linker is more conformationally restrained [25]. The 1,4-diamino-butane linker **1** was treated with di-*tert*-butyldicarbonate (Boc_2_O) in 1,4-dioxane at room temperature to afford mainly the mono-*N*-Boc protected amine **2** in good yield (84%) [26]. To obtain a sufficient number of compounds suitable for a preliminary biological screening, we privileged the transformation of *N*-Boc-1,4-diamino-butane **2** into mono protected *N*-arylmethyl diamine **5** by reductive amination in two steps. The (4-arylmethylamino)butyl chain appended on the *N*-3 position of the 2-thioxo-1,3-thiazolidine-4-one platform represent the first point of diversity for the desired target compounds **9**. Preparation of **4** was easily realized by reaction between of appropriate arylaldehyde **3a**–**f** with *N*-Boc diamine **2** in the presence of molecular sieves 3Å and, the condensation was conducted in a solution of diethyl ether Et_2_O at room temperature during 24 h. Then, transformation of arylaldimines **4** into mono protected *N*-arylmethyl diamines **5** could be readily accomplished in good yields (85% to 98%) using NaBH_4_ (5 equiv.) in MeOH at 50 °C during 24 h. For the cleavage of *N*-Boc group, the use of trifluoroacetic acid in dichloromethane is often efficient, but in our case, we observed the formation of impurities resulting from uncontrolled degradation of **5** in this strong acidic media. We thus preferred to use a more classical and practical approach by using a solution of 6M HCl. The deprotection was conducted in 1,4-dioxane at room temperature after 4 h of reaction time. As can be seen from the data presented in Table 1, the *N*-(arylmethyl)butane-1,4-diamine hydrochloride **6a**–**f** were efficiently prepared with electron-rich and electron-poor aldehydes **3a**–**f** in yields ranging from 72% to 98%.

With the desired salts **6** in hand, we wished to examine the construction of the 2-thioxo-1,3-thiazolidine-4-one platform followed by Knoevenagel condensation using arylaldehyde **3** under microwave irradiation for installation of the 5-arylidene moiety. The choice of arylaldehydes **3** in Knoevenagel condensation represent, in fact, the second point of diversity in the final structure of the targeted compounds **9**. In a previous work issued from our laboratory [23], we have developed a “one-pot two-steps” method under microwave irradiation, which was applied for the synthesis of unsymmetrical linked bis-arylidene rhodanines from symmetric diamines.

**Table 1:** Results for the preparation of compounds **2**, **4**, **5**, **6** and **9**.

Compound	Starting Product	Reaction Conditions under MWI *^a^*				Yield *^b^* (%)
		Starting Product 6	Number of equiv. for Et_3_N	Reaction Time for 8 *^c^* (min)	Reaction Time for Condensation *^d^* (min)	
**2**	-	-	-	-	-	84
**4a**	**3a**	-	-	-	-	97
**4b**	**3b**	-	-	-	-	99
**4c**	**3c**	-	-	-	-	98
**4d**	**3d**	-	-	-	-	98
**4e**	**3e**	-	-	-	-	98
**4f**	**3f**	-	-	-	-	99
**5a**	**4a**	-	-	-	-	93
**5b**	**4b**	-	-	-	-	99
**5c**	**4c**	-	-	-	-	85
**5d**	**4c**	-	-	-	-	90
**5e**	**4e**	-	-	-	-	97
**5f**	**4f**	-	-	-	-	98
**6a**	**5a**	-	-	-	-	84
**6b**	**5b**	-	-	-	-	97
**6c**	**5c**	-	-	-	-	98
**6d**	**5d**	-	-	-	-	98
**6e**	**5e**	-	-	-	-	72
**6f**	**5f**	-	-	-	-	92
**9a**	**3e**	**6b**	3	30	30	10
**9b**	**3b**	**6b**	3	30	30	6
**9c**	**3f**	**6b**	3	30	30	5
**9d**	**3g**	**6b**	3	30	30	30
**9e**	**3e**	**6d**	3	15	15	21
**9f**	**3f**	**6d**	3	15	15	9
**9g**	**3d**	**6d**	3	30	30	5
**9h**	**3e**	**6e**	2	15	15	59
**9i**	**3f**	**6e**	2	15	15	59
**9j**	**3h**	**6e**	2	15	15	34
**9k**	**3g**	**6e**	3	30	30	46
**9l**	**3e**	**6f**	3	15	15	15
**9m**	**3f**	**6f**	3	15	15	7
**9n**	**3g**	**6f**	3	30	30	34

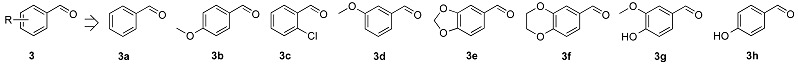
Notes: *^a^* Reaction realized in a tube (sealed with a snap cap) under microwave irradiation (μω) with the Monowave^®^ 300 Anton-Paar reactor. *^b^* Isolated yield. *^c^* Reaction temperature: 90 °C for the preparation of **8** (1st period of microwave irradiation). *^d^* Reaction temperature: 110 °C for condensation reaction after addition of **3** (2nd period of microwave irradiation).

Application of this methodology, based on a “modified Holmberg method”, first involved, in our case, the reaction of the commercial bis-(carboxymethyl)-trithiocarbonate reagent **7** with the synthesized *N*-(arylmethyl)butane-1,4-diamine hydrochloride **6** to afford the intermediate **8** and secondly, Knoevenagel condensation to produce the desired 5-arylidene rhodanines **9**. This “one-pot two-steps” methodology was realized under microwave dielectric heating because the use of commercial scientific laboratory microwave apparatus favoured higher product yields and significant rate enhancements compared to reactions which run with conventional heating (*i.e.*, in oil bath) [27]. After several experiments, optimal reaction conditions for this “one-pot two-steps” synthesis involved reaction of a stoichiometric mixture of *N*-(arylmethyl)butane-1,4-diamine hydrochloride **6** and commercial reagent **7** solubilized in dimethoxyethane with one or two equivalents of triethylamine. This starting reaction mixture was placed in a commercial glass tube closed with a snap cap and was irradiated with appropriate reaction time (15 or 30 min.) at 90 °C. After this first period of microwave irradiation followed by a cooling down to room temperature, aromatic aldehyde **3** was directly added to the crude suspension and was submitted again to microwave dielectric heating at 110 °C during 15 or 30 min for condensation.

The desired compound **9** was obtained as precipitate after addition of methanol in the solventless crude reaction mixture (after elimination of the volatile compounds *in vacuo*) and triturated, followed by successive washings with deionized water, ethanol, ether and finally was recrystallized from absolute EtOH to increase the quality of the precipitated product **9**. A set of 14 pure compounds **9a**–**n** was prepared in 5%–59% yield (Table 1) and all the products **9** were characterized by ^1^H-, ^13^C-NMR and HRMS before entering the biological tests. For the exocyclic double bond (CH=C) in C-5 position of all the 3-(4-arylmethylamino)butyl-2-thioxo-1,3-thiazolidine-4-one **9a**–**n**, it’s possible in theory to observe *E-* and/or *Z-*geometrical isomers. Examination of their ^1^H-NMR spectra in DMSO-*d*_6_ showed only one signal for the methylene proton (CH=) in the range 7.54–7.74 ppm at lower field values than those expected for the *E-*isomers, which indicates that all the compounds **9a**–**n** have the *Z*-configuration due to the high degree of thermodynamic stability of this isomer [28,29]. In ^13^C-NMR, the signal of C-5 (C=) appears in the range 120.0–127.5 ppm, and we also observed only one signal for the exocyclic methylene proton (134.6 < δ_CH=_ < 135.9 ppm) that confirmed the presence of only the *Z-*geometrical isomer for the targeted compounds **9a**–**n**.

### 2.2. Biology

**Figure 2:**
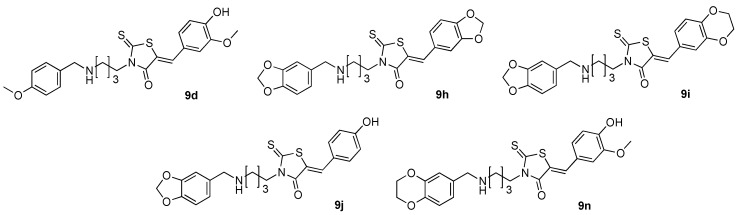
Structure of compounds **9d**, **9**(**h**–**j**) and **9n**, which are active against protein kinases and/or tumor cell lines.

As an initial effort to investigate their *in vitro* bioactivity, the new synthesized 3-(4-arylmethylamino)butyl-2-thioxo-1,3-thiazolidine-4-one **9a**–**n** and also their precursors **6a**–**f** were evaluated for their *in vitro* inhibition of cell proliferation. For this study, we used a panel of seven representative tumoral cell lines, namely HuH7 D12 (differential hepato cellular carcinoma), Caco 2 (differentiating colorectal adenocarcinoma), MDA-MBD231 (prostate carcinoma), HCT 116 (actively proliferating colorectal adenocarcinoma), PC3 (prostate carcinoma), NCI-H727 (lung carcinoma), HaCat keratinocyte and, diploid skin fibroblasts as normal cell lines for control. Roscovitine, Doxorubicine and Taxol were also used as positive controls and their IC_50_ values are compared with those obtained for compounds **6** and **9**. Results of the *in vitro* antiproliferative data activity are reported in Table 2. None of the *N*-(arylmethyl)butane-1,4-diamines hydrochloride **6a**–**f** presented a significant activity against the seven representative tumoral cell lines. For the other compounds **9**, the most active compound was clearly **9i** (Figure 2) and exhibited antitumor activities in the HuH7 D12, MDA-MBD231 and HaCat cell lines with IC_50_ values lower than 10 μM (HuH7 D12 and MDA-MBD231: IC_50_ 9 μM) and did not inhibit the growth of normal fibroblasts (IC_50_ > 25 μM). In addition, compounds **9d**, **9h**, **9j** and **9n** presented moderate antitumor activities (IC_50_ 10–17 μM) in all cell lines of the panel but without selectivity.

**Table 2:** Antiproliferative activity of compounds **6**(**a**–**e**) and **9**(**a**–**n**) on six representative tumor cell lines.

Compound	% of Survival *^a^* and IC_50_ (μM) of Selected Compounds *^b^*							
	Huh7 D12	Caco 2	MDA-MB231	HCT 116	PC3	NCI-H727	HaCat	Fibroblasts
**6a**	102	114	104	112	100	97	97	93
**6b**	107	114	100	112	100	86	106	100
**6c**	82	93	102	98	96	100	80	84
**6d**	107	111	99	111	98	92	96	93
**6e**	91	106	102	102	96	98	96	93
**6f**	88	109	99	96	97	92	94	105
**9a**	85	109	100	109	92	104	103	104
**9b**	105	108	97	102	99	90	104	167
**9c**	88	93	92	97	97	95	101	102
**9d**	7 (10)	23 (15)	15 (11)	1 (10)	41 (17)	11 (10)	2 (10)	29 (>25)
**9e**	74	97	97	99	85	102	86	122
**9f**	102	110	98	113	98	96	100	134
**9g**	111	117	104	118	98	95	110	100
**9h**	7 (10)	42 (14)	49 (10)	65 (13)	51 (13)	43 (>25)	10 (10)	80 (>25)
**9i**	5 (9)	17 (13)	17 (9)	8 (14)	39 (14)	9 (11)	6 (8)	54 (>25)
**9j**	22 (11)	41 (27)	42 (19)	40 (22)	38 (19)	33 (19)	21 (13)	77 (>25)
**9k**	72	98	92	98	83	96	80	84
**9l**	90	105	98	106	93	96	95	128
**9m**	97	107	99	103	96	98	94	149
**9n**	41 (15)	28 (16)	55 (15)	52 (21)	61 (19)	63 (17)	48 (15)	86 (>25)
Roscovitine	21 (15)	3 (15)	21 (12	10 (9)	24 (13)	30 (43)	6 (11)	53 (125)
Doxorubicine	63 (0.03)	43 (0.03)	82 (0.01)	22 (0.03)	34	65	88 (0.02)	74 (>0.25)
Taxol	36 (0.003)	29 (0.04)	41 (0.04)	9 (<0.001)	35 (<0.001)	59	11 (0.001)	76 (>25)
DMSO	100 (>25)	100 (>25)	100 (>25)	100 (>25)	100 (>25)	100 (>25)	100 (>25)	100 (>25)

Notes: *^a^* Percentage of survival measured at 25 μM (after 48 h using a single dose, triplicate). *^b^* IC_50_ values in brackets are expressed in μM and are the average of three assays, standard error ± 0.5 μM.

Then, we evaluated the intermediates salts **6a**–**f** and fourteen final compounds **9a**–**n** on four protein kinases: *Hs*CDK5-p25 (cyclin-dependant kinase 5-p25), *Ss*GSK-3α/β (glycogen synthase kinase-3α/β) *Ss*CK1δ/ε (Caseine kinase 1) and *Hs*Haspin as an essential mitotic kinase [30]. All assays were run in the presence of 15 μM of ATP and appropriate substrates. IC_50_ values were determined from dose-response curves. Concerning the library of the fourteen (5*Z*) 3-(4-arylmethylamino)butyl-5-arylidene-2-thioxo-1,3-thiazolidine-4-ones **9a**–**n** and also the six diamines salts **6a**–**f**, the results are given in Table 3. Among all the products, the main part of their inhibition activity was focused on two proteins kinases (*Ss*CK1 and *Hs*CDK5-p25) for four products, which exhibited inhibitory activity in the micromolar range (*Ss*CK1: 1.4 μM < IC_50_ < 6.6 μM; *Hs*CDK5-p25: IC_50_ 1.2 μM). For the compound **9j** and **9n**, we observed micromolar inhibition activity on *Ss*CK1 (IC_50_ values for **9j**: 1.4 μM and **9n**: 2 μM) with good selectivity. It is interesting to note that these two compounds are respectively substituted with an hydroxyl function on *para*-position of the 5-arylidene moiety and also with a similar bulky group (1,3-benzodioxol-5-yl for **9j** and 2,3-dihydro-benzo[1,4]dioxin-6-yl for **9n**) on the arylmethylamino moiety grafted on the *N*-3 butyl chain. On the contrary, the presence of 1,3-benzodioxol-5-yl (for **9h**) or 2,3-dihydro-benzo[1,4]dioxin-6-yl moiety (for **9i**) in the 5-arylidene group induced a moderate loss of inhibition activity for *Ss*CK1 (IC_50_ for **9h**: 6.6 μM and **9i**: 5.4 μM) associated with micromolar affinity for *Hs*CDK5-p25 (IC_50_ for **9h**: 1.1 μM and **9i**: 1.3 μM). Compound **9i** presented also inhibition proliferation activity on Huh7 D12, MDA-MBD231 cell lines and is inactive against normal fibroblasts.

**Table 3:** Effects of the compounds **6a**–**f** and **9a**–**n** on the catalytic activity of four purified protein kinases *^a^*.

Compound	*Hs*CDK5/p25	*Ss*GSK-3α/β	*Ss*CK1δ/ε	*Hs*Haspin
**6a–f**	>10	>10	>10	>10
**9a–g**	>10	>10	>10	>10
**9h**	1.1	>10	6.6	>10
**9i**	1.3	>10	5.1	>10
**9j**	>10	>10	1.4	>10
**9k–m**	>10	>10	>10	>10
**9n**	10	>10	2	>10

Note: *^a^* Compounds were tested at various concentrations on each kinase as described in Experimental Section. IC_50_ values, calculated from the dose-response curves, are reported in μM > 10, inhibitory but IC_50_ > 10 μM.

## 3. Experimental Section

### 3.1. Chemistry Section

#### 3.1.1. General Section

Melting points were determined on a Kofler melting point apparatus and were uncorrected. Thin-layer chromatography (TLC) was accomplished on 0.2-mm precoated plates of silica gel 60 F-254 (Merck, Fontenay-sous-Bois, France). Visualization was made with ultraviolet light (254 and 365 nm) or with a fluorescence indicator. ^1^H-NMR spectra were recorded on BRUKER AC 300 P (300 MHz) spectrometer, ^13^C-NMR spectra on a BRUKER AC 300 P (75 MHz) spectrometer. Chemical shifts are expressed in parts per million downfield from tetramethylsilane as an internal standard. Data are given in the following order: δ value, multiplicity (s, singlet; d, doublet; t, triplet; q, quartet; m, multiplet; br, broad), number of protons, coupling constants *J* is given in Hertz. The mass spectra (HRMS) were taken respectively on a MS/MS ZABSpec Tof Micromass (EBE TOF geometry) at an ionizing potential of 8 eV and on a VARIAN MAT 311 at an ionizing potential of 70 eV in the Centre Régional de Mesures Physiques de l’Ouest (CRMPO, Rennes, France). Reactions under microwave irradiations were realized in the Anton Paar Monowave 300^®^ microwave reactor (Anton-Paar, Courtaboeuf, France) using borosilicate glass vials of 10 mL equipped with snap caps (at the end of the irradiation, cooling reaction was realized by compressed air). The microwave instrument consists of a continuous focused microwave power output from 0 to 800 W for this Monowave 300^®^ apparatus. All the experiments in the microwave reactor were performed using a stirring option. The target temperature was reached with a ramp of 5 min and the chosen microwave power stayed constant to hold the mixture at this temperature. The reaction temperature is monitored using calibrated infrared sensor and the reaction time included the ramp period. The microwave irradiation parameters (power and temperature) were monitored by the Monowave software package of the Monowave 300^®^ microwave reactor. Solvents were evaporated with a BUCHI rotary evaporator. All reagents and solvents were purchased from Acros, Sigma-Aldrich Chimie (Saint-Quentin Fallavier, France), TCI France and Fluka France and were used without further purification.

*tert-Butyl (4-aminobutyl)carbamate* (**2**). In a 250 mL two-necked round-bottomed flask, provided with magnetic stirrer and condenser, commercial 1,4-diaminobutane **1** (14.3 mL, 12.5 g, 0.14 mol) was solubilized in 69 mL of 1,4-dioxane at room temperature. To this mixture was added dropwise a solution of commercial di-*tert*-butyldicarbonate (6.5 g, 30 mmol) in 1,4-dioxane (85 mL) over a period of 3 h. After vigorous stirring at 25 °C during a 12 h period, the volatile compounds of the reaction mixture were eliminated *in vacuo* and into the crude reaction mixture was poured 150 mL of deionized water. The mixture was extracted with methylene chloride (5 × 50 mL), organic phases were collected and dried over magnesium sulfate. The filtrate was concentrated in a rotary evaporator under reduced pressure and was dried under high vacuum (10^−2^ Torr) at 25 °C for 10 min. The desired carbamate **2** (1.58 g) was obtained as a colourless mobile oil in 84% yield and was further used without purification. ^1^H-NMR (DMSO-*d*_6_) δ: 1.34 (s, 9H, Me_3_CO); 1.40 (m, 4H, CH_2_); 2.61 (t, 2H, *J* = 6.7 Hz, CH_2_); 3.02 (m, 2H, CH_2_NH); 4.93 (br s, 2H, NH**_2_**); 5.71 (br s, 1H, NH). ^13^C-NMR (DMSO-*d*_6_) δ: 27.2 (CH_2_); 28.4 (OC(CH_3_)_3_); 29.5 (CH_2_); 40.2 (CH_2_); 41.1 (CH_2_); 78.9 (OC(CH_3_)_3_); 156.1 (C=O). HRMS, *m*/*z*: 189.1600 found (calculated for C_9_H_21_N_2_O_2_ [M + H]^+^ requires 189.1603).

#### 3.1.2. Standard Procedure for the Preparation of Aldimines **4a**–**f** from *tert*-Butyl (4-aminobutyl)Carbamate **2** and Aromatic Aldehyde **3a**–**f**

In a 100 mL two-necked round-bottomed flask, provided with magnetic stirrer and condenser, containing a solution of *tert*-butyl (4-aminobutyl)carbamate **2** (1.97 g, 10.5 mmol) in anhydrous ether (50 mL) was added dropwise during 30 min, a suspension of commercial aldehyde (10 mmol) in anhydrous ether (30 mL) and molecular sieves (2 g, 3 Å, 8–12 mesh). The crude reaction mixture is stirred vigorously for 16 h at room temperature until the disappearance of aromatic aldehyde **3** controlled by thin-layer chromatography on 0.2-mm precoated plates of silica gel 60 F-254 (Merck). The crude reaction mixture was filtered on filter paper and then concentrated in a rotary evaporator under reduced pressure. The crude residue was dried under high vacuum (10^−2^ Torr) at 25 °C for 10 min. The desired aldimine **4** was obtained as yellowish viscous oil and was further used without purification.

*[4-(Benzylidene-amino)-butyl]-carbamic acid tert-butyl ester* (**4a**). Compound **4a** was prepared in 97% yield (2.68 g) from benzaldehyde **3a** (1.06 g, 10 mmol) according to the standard procedure as yellowish viscous oil. ^1^H-NMR (DMSO-*d*_6_) δ: 1.37 (s, 9H, Me_3_C); 1.40–1.48 (m, 2H, CH_2_, H-2); 1.54–1.62 (m, 2H, CH_2_, H-3); 2.96 (q, 2H, *J* = 6.8 Hz, CH_2_NH, H-1); 3.55 (t, 2H, *J* = 6.8 Hz, CH_2_N=, H-4); 6.79–6.83 (br s, 1H, NH); 7.41–7.46 (m, 3H, H-3ʹ, H-4ʹ, H-5ʹ, Ar); 7.71–7.75 (m, 2H, H-2ʹ, H-6ʹ, Ar); 8.32 (s, 1H, N=CH). ^13^C-NMR (DMSO-*d*_6_) δ: 27.3 (C-2); 27.8 (C-3); 28.2 (OC(CH_3_)_3_); 39.6 (C-1); 60.1 (C-4); 77.2 (OC**(**CH_3_)_3_); 127.7 (C-2ʹ, C-6ʹ, Ar); 128.5 (C-3ʹ, C-5ʹ, Ar); 130.4 (C-4ʹ, Ar); 136.1 (C-1ʹ, Ar); 155.5 (C=O); 160.5 (N=CH).

*{4-[(4-Methoxy-benzylidene)-amino]-butyl}-carbamic acid tert-butyl ester* (**4b**). Compound **4b** was prepared in 99% yield (3.03 g) from 4-methoxybenzaldehyde **3b** (1.36 g, 10 mmol) according to the standard procedure as yellowish viscous oil. ^1^H-NMR (DMSO-*d*_6_) δ: 1.37 (s, 9H, Me_3_C); 1.32–1.46 (m, 2H, CH_2_, H-2); 1.52–1.61 (m, 2H, CH_2_, H-3); 2.94 (q, 2H, *J* = 6.9 Hz, CH_2_NH, H-1); 3.50 (t, 2H, *J* = 6.8 Hz, CH_2_N=, H-4); 3.80 (s, 3H, OCH_3_); 6.77–6.82 (br s, 1H, NH); 6.98 (d, 2H, *J* = 8.8 Hz, H-2ʹ, H-6ʹ, Ar); 7.66 (d, 2H, *J* = 8.8 Hz, H-3ʹ, H-5ʹ, Ar); 8.24 (s, 1H, N=CH). ^13^C-NMR (DMSO-*d*_6_) δ: 27.4 (C-2); 28.0 (C-3); 28.2 (OC(CH_3_)_3_); 39.6 (C-1); 55.2 (OCH_3_); 60.1 (C-4); 77.3 (OC**(**CH_3_)_3_); 114.0 (C-2ʹ, C-6ʹ, Ar); 129.0 (C-1ʹ, Ar); 129.3 (C-3ʹ, C-5ʹ, Ar); 155.5 (C=O); 159.7 (N=CH); 161.0 (C-4ʹ, Ar).

*{4-[(2-Chloro-benzylidene)-amino]-butyl}-carbamic acid tert-butyl ester* (**4c**). Compound **4c** was prepared in 98% yield (3.05 g) from 2-chlorobenzaldehyde **3c** (1.41 g, 10 mmol) according to the standard procedure as yellowish viscous oil. ^1^H-NMR (DMSO-*d*_6_) δ: 1.37 (s, 9H, Me_3_C); 1.34–1.48 (m, 2H, CH_2_, H-2); 1.56–1.63 (m, 2H, CH_2_, H-3); 2.96 (q, 2H, *J =* 6.7 Hz, CH_2_NH, H-1); 3.61 (t, 2H, *J =* 6.8 Hz, CH_2_N=, H-4); 6.78–6.82 (br s, 1H, NH); 7.36–7.52 (m, 3H, H-4ʹ, H-5ʹ, H-6ʹ, Ar); 7.94–7.97 (m, 1H, H-3ʹ, Ar); 8.65 (s, 1H, N=CH). ^13^C-NMR (DMSO-*d*_6_) δ: 27.3 (C-2); 27.7 (C-3); 28.2 (OC(CH_3_)_3_); 39.6 (C-1); 60.4 (C-4); 77.2 (OC(CH_3_)_3_); 127.4 (C-5ʹ, Ar); 128.0 (C-4ʹ, Ar); 129.8 (C-6ʹ, Ar); 132.0 (C-3ʹ, Ar); 132.7 (C-1ʹ, C-2ʹ, Ar); 133.8 (C-1ʹ, C-2ʹ, Ar); 155.5 (C=O); 156.6 (N=CH).

*{4-[(3-Methoxy-benzylidene)-amino]-butyl}-carbamic acid tert-butyl ester* (**4d**). Compound **4d** was prepared in 98% yield (3.03 g) from 3-methoxybenzaldehyde **3d** (1.36 g, 10 mmol) according to the standard procedure as yellowish viscous oil. ^1^H-NMR (DMSO-*d*_6_) δ: 1.37 (s, 9H, Me_3_C); 1.36–1.45 (m, 2H, CH_2_, H-2); 1.54–1.64 (m, 2H, CH_2_, H-3); 2.96 (q, 2H, *J =* 6.7 Hz, CH_2_NH, H-1); 3.54 (t, 2H, *J =* 6.7 Hz, CH_2_N=, H-4); 3.78 (s, 3H, OCH_3_), 6.76–6.82 (br s, 1H, NH); 6.99–7.03 (m, 1H, H-5ʹ, Ar); 7.27–7.38 (m, 3H, H-2ʹ, H-4ʹ, H-6ʹ, Ar); 8.29 (s, 1H, N=CH). ^13^C-NMR (DMSO-*d*_6_) δ: 27.3 (C-2); 27.8 (C-3); 28.2 (OC(CH_3_)_3_); 39.6 (C-1); 55.0 (OCH_3_); 60.1 (C-4); 77.2 (OC(CH_3_)_3_); 111.9 (C-5ʹ, Ar); 116.6 (C-6ʹ, Ar); 120.6 (C-4ʹ, Ar); 129.6 (C-2ʹ, Ar); 137.6 (C-1ʹ, Ar); 155.5 (C=O); 159.4 (C-3ʹ, Ar); 160.4 (C=N).

*{4-[(Benzo[1,3]dioxol-5-ylmethylene)-amino]-butyl}-carbamic acid tert-butyl ester* (**4e**). Compound **4e** was prepared in 98% yield (3.13 g) from 3,4-methylenedioxybenzaldehyde **3e** (1.51 g, 10 mmol) according to the standard procedure as yellowish viscous oil. ^1^H-NMR (DMSO-*d*_6_) δ: 1.37 (s, 9H, Me_3_C); 1.30–1.46 (m, 2H, CH_2_, H-2); 1.51–1.61 (m, 2H, CH_2_, H-3); 2.94 (q, 2H, *J =* 6.9 Hz, CH_2_NH, H-1); 3.49 (t, 2H, *J =* 6.7 Hz, CH_2_N=, H-4); 6.07 (s, 2H, H-2ʹ, Ar), 6.77–6.82 (br s, 1H, NH); 6.97 (d, 1H, *J =* 8 Hz, H-6ʹ, H-7ʹ, Ar); 7.16–7.20 (m, 1H, H-6ʹ, H-7ʹ, Ar); 7.27–7.28 (m, 1H, H-4ʹ, Ar), 8.20 (s, 1H, N=CH). ^13^C-NMR (DMSO-*d*_6_) δ: 27.3 (C-2); 27.9 (C-3); 28.2 (OC(CH_3_)_3_); 39.6 (C-1); 59.8 (C-4); 77.4 (OC(CH_3_)_3_); 101.4 (C-2ʹ, Ar); 105.8 (C-6ʹ, Ar); 108.1 (C-7ʹ, Ar); 124.0 (C-4ʹ, Ar); 130.9 (C-5ʹ, Ar); 147.8 (C-3aʹ, C-7aʹ, Ar); 149.3 (C-3aʹ, C-7aʹ, Ar); 155.5 (C=O); 160.0 (C=N).

*{4-[(2,3-Dihydro-benzo[1,4]dioxin-6-ylmethylene)-amino]-butyl}-carbamic acid tert-butylester* (**4f**). Compound **4f** was prepared in 99% yield (3.31 g) from 2,3-dihydro-benzo[1,4]dioxin-6-carbaldehyde **3f** (1.50 g, 10 mmol) according to the standard procedure as yellowish viscous oil. ^1^H-NMR (DMSO-*d*_6_) δ: 1.37 (s, 9H, Me_3_C); 1.34–1.45 (m, 2H, CH_2_, H-2); 1.51–1.60 (m, 2H, CH_2_, H-3); 2.94 (q, 2H, *J =* 6.8 Hz, CH_2_NH, H-1); 3.50 (t, 2H, *J =* 6.7 Hz, CH_2_N=, H-4); 4.23–4.30 (m, 4H, H-2ʹ, H-3ʹ, Ar), 6.77–6.81 (br s, 1H, NH); 6.88–6.91 (m, 1H, H-7ʹ, Ar); 7.18–7.21 (m, 2H, H-5ʹ, H-8ʹ, Ar); 8.17 (s, 1H, N=CH). ^13^C-NMR (DMSO-*d*_6_) δ: 27.3 (C-2); 27.9 (C-3); 28.2 (OC(CH_3_)_3_); 39.6 (C-1); 60.0 (C-4); 63.9 (C-2ʹ, Ar), 64.2 (C-3ʹ, Ar), 77.2 (OC(CH_3_)_3_); 116.0 (C-7ʹ, Ar); 117.1 (C-8ʹ, Ar); 121.2 (C-5ʹ, Ar); 129.8 (C-6ʹ, Ar); 143.4 (C-4aʹ, C-8aʹ, Ar); 145.4 (C-4aʹ, C-8aʹ, Ar); 155.5 (C=O); 159.6 (C=N).

#### 3.1.3. Standard Procedure for Reduction of Aldimines **4a**–**f** into *N*-Boc Monoprotected Diamines **5a**–**f**

In a 50 mL two-necked round-bottomed flask, provided with a magnetic stirrer and reflux condenser, compound **4** (5 mmol) was dissolved in methanol p.a. (30 mL) under vigorous stirring and cooled at 0 °C. To this solution was added by small portions commercial sodium borohydride NaBH_4_ (0.95 g, 25 mmol) over a period of 20 min. The resulting suspension was stirred at 50 °C for 24 h (monitored by TLC on 0.2-mm precoated plates of silica gel 60 F-254, Merck). After cooling down to room temperature, the volatile compounds were removed in a rotary evaporator under reduced pressure, then deionized water (40 mL) was added in one portion to the crude residue. The mixture was transferred to a separating funnel and was extracted with dichloromethane (3 × 50 mL). The combined organic phases were dried over magnesium sulphate MgSO_4_, filtered on filter paper and the solvent was eliminated *in vacuo*. The crude residue was dried under high vacuum (10^−2^ Torr) at 25 °C for 2 h. The desired compound **5** was obtained as yellowish viscous oil and was further used without purification.

*(4-Phenylmethylamino-butyl)-carbamic acid tert-butyl ester* (**5a**). Compound **5a** was prepared in 93% yield (1.25 g) from [4-(benzylidene-amino)-butyl]-carbamic acid *tert*-butyl ester **4a** (1.38 g, 5 mmol) according to the standard procedure that gave **5a** as yellowish viscous oil. ^1^H-NMR (DMSO-*d*_6_) δ: 1.37 (s, 9H, Me_3_C); 1.34–1.42 (m, 4H, CH_2_, H-2, H-3); 2.45 (t, 2H, *J* = 6.2 Hz, CH_2_NH, H-4); 2.89–2.91 (m, 2H, CH_2_NH, H-1); 3.66 (s, 2H, ArCH_2_NH); 6.78–6.82 (br s, 1H, NHCO); 7.18–7.13 (m, 1H, H-4ʹ, Ar); 7.26–7.33 (m, 4H, H-2ʹ, H-3ʹ, H-5ʹ, H-6ʹ, Ar). ^13^C-NMR (DMSO-*d*_6_) δ: 27.4 (C-3); 26.8 (C-2); 39.8 (C-1); 48.4 (C-4); 53.0 (ArCH_2_NH); 77.2 (OC(CH_3_)_3_); 126.3 (C-4ʹ, Ar); 127.8 (C-2ʹ, C-6ʹ, Ar); 128.0 (C-3ʹ, C-5ʹ, Ar); 141,0 (C-1ʹ, Ar); 155,5 (C=O).

*[4-(4-Methoxy-phenylmethylamino)-butyl]-carbamic acid tert-butyl ester* (**5b**). Compound **5b** was prepared in 99% yield (1.52 g) from {4-[(4-methoxy-benzylidene)-amino]-butyl}-carbamic acid *tert-*butyl ester **4b** (1.53 g, 5 mmol) according to the standard procedure that gave **5b** as yellowish viscous oil. ^1^H-NMR (DMSO-*d*_6_) δ: 1.37 (s, 9H, Me_3_C); 1.33–1.41 (m, 4H, H-2, H-3); 2.45 (t, 2H, *J* = 6.5 Hz, CH_2_NH, H-4); 2.88–2.90 (m, 2H, CH_2_NHCO, H-1); 3.60 (s, 2H, ArCH_2_NH); 3.72 (s, 3H, OCH_3_); 6.76–6.80 (br s, 1H, NHCO); 6.85 (d, 2H, *J* = 8.5 Hz, H-2ʹ, H-6ʹ, Ar); 7.22 (d, 2H, *J* = 8.6 Hz, H-3ʹ, H-5ʹ, Ar). ^13^C-NMR (DMSO-*d*_6_) δ: 26.6 (C-2); 27.4 (C-3); 28.2 (OC(CH_3_)_3_); 39.8 (C-4); 48.2 (C-1); 52.3 (ArCH_2_NH); 54.9 (OCH_3_); 77.2 (OC(CH_3_)_3_); 113.4 (C-2ʹ, C-6ʹ, Ar); 129.0 (C-3ʹ, C-5ʹ, Ar); 132.6 (C-1ʹ, Ar); 155.5 (C=O); 158.0 (C-4ʹ, Ar).

*[4-(2-Chloro-phenylmethylamino)-butyl]-carbamic acid tert-butyl ester* (**5c**). Compound **5c** was prepared in 85% yield (1.33 g) from {4-[(2-chloro-benzylidene)-amino]-butyl}-carbamic acid *tert-*butyl ester **4c** (1.55 g, 5 mmol) according to the standard procedure that gave **5c** as yellowish viscous oil. ^1^H-NMR (DMSO-*d*_6_) δ: 1.37 (s, 9H, Me_3_C); 1.34–1.43 (m, 4H, CH_2_, H-2, H-3, 4H); 2.47–2.52 (m, 2H, ArCH_2_NH, H-4); 2.88–2.92 (m, 2H, CH_2_NHCO, H-1); 3.75 (s, 2H, ArCH_2_NH); 6.77–6.80 (br s, 1H, NHCO); 7.21–7.33 (m, 2H, H-4ʹ, H-5ʹ, Ar); 7.37–7.4 (m, 1H, H-6ʹ, Ar); 7.5–7.53 (m, 1H, H-3ʹ, Ar). ^13^C-NMR (DMSO-*d*_6_) δ: 26.7 (C-2); 27.4 (C-3); 28.2 (OC(CH_3_)_3_); 39.8 (C-4); 48.5 (C-1); 50.0 (ArCH_2_NH); 77.2 (OC(CH_3_)_3_); 126.9 (C-5ʹ, Ar); 128.0 (C-4ʹ, Ar); 128.9 (C-6ʹ, Ar); 129.6 (C-3ʹ, Ar); 132.5 (C-1ʹ, Ar); 138.2 (C-2ʹ, Ar); 155.5 (C=O).

*[4-(3-Methoxy-phenylmethylamino)-butyl]-carbamic acid tert-butyl ester* (**5d**). Compound **5d** was prepared in 90% yield (1.39 g) from {4-[(3-methoxy-benzylidene)-amino]-butyl}-carbamic acid *tert-*butyl ester **4d** (1.53 g, 5 mmol) according to the standard procedure that gave **5d** as pale pink viscous oil. ^1^H-NMR (DMSO-*d*_6_) δ: 1.37 (s, 9H, Me_3_C); 1.34–1.46 (m, 4H, H-2, H-3); 2.45 (t, 2H, *J* = 6.4 Hz, CH_2_NH, H-4); 2.89–2.91 (m, 2H, CH_2_NHCO, H-1); 3.64 (s, 2H, ArCH_2_NH); 3.74 (s, 3H, OCH_3_); 6.75–6.80 (br s, 1H, NHCO); 6.75–6.91 (m, 3H, H-4ʹ, H-5ʹ, H-6ʹ, Ar); 7.17–7.22 (m, 1H, H-2ʹ, Ar). ^13^C-NMR (DMSO-*d*_6_) δ: 26.8 (C-2); 27.4 (C-3); 28,2 (OC(CH_3_)_3_); 39.8 (C-4); 48.4 (C-1); 52.9 (ArCH_2_NH); 54.8 (OCH_3_); 77.2 (OC(CH_3_)_3_); 111.8 (C-5ʹ, Ar); 113.2 (C-6ʹ, Ar); 120.0 (C-4ʹ, Ar); 128.9 (C-2ʹ, Ar); 142.8 (C-1ʹ, Ar); 155.5 (C=O); 159.2 (C-3ʹ, Ar).

*{4-[(Benzo[1,3]dioxol-5-ylmethyl)-amino]-butyl}-carbamic acid tert-butyl ester* (**5e**). Compound **5e** was prepared in 97% yield (1.56 g) from {4-[(benzo[1,3]dioxol-5-ylmethylene)-amino]-butyl}-carbamic acid *tert-*butyl ester **4e** (1.60 g, 5 mmol) according to the standard procedure that gave **5e** as yellowish viscous oil. ^1^H-NMR (DMSO-*d*_6_) δ: 1.37 (s, 9H, Me_3_C); 1.23–1.40 (m, 4H, H-2, H-3); 2.43 (t, 2H, *J* = 6.5 Hz, CH_2_NH, H-4); 2.89–2.91 (m, 2H, CH_2_NHCO, H-1); 3.64 (s, 2H, ArCH_2_NH); 5.96 (s, 2H, H-2ʹ, Ar); 6.73–6.82 (br s, 1H, NHCO); 6.73–6.90 (m, 3H, H-4ʹ, H-6ʹ, H-7ʹ, Ar). ^13^C-NMR (DMSO-*d*_6_) δ: 26.8 (C-2); 27.4 (C-3); 28.2 (OC(CH_3_)_3_); 39.8 (C-4); 48.1 (C-1); 52.7 (ArCH_2_NH); 77.2 (OC(CH_3_)_3_); 100.6 (C-2ʹ, Ar); 107.7 (C-6ʹ, Ar); 108.2 (C-7ʹ, Ar); 120.8 (C-4ʹ, Ar); 135.0 (C-5ʹ, Ar); 145.7 (C-3aʹ, C-7aʹ, Ar); 147.1 (C-3aʹ, C-7aʹ, Ar); 155.5 (C=O).

*{4-[(2,3-Dihydro-benzo[1,4]dioxin-6-ylmethyl)-amino]-butyl}-carbamic acid tert-butyl ester* (**5f**). Compound **5f** was prepared in 88% yield (1.48 g) from {4-[(2,3-dihydro-benzo[1,4]dioxin-6-ylmethylene)-amino]-butyl}-carbamic acid *tert-*butylester **4f** (1.67 g, 5 mmol) according to the standard procedure that gave **5f** as yellowish viscous oil. ^1^H-NMR (DMSO-*d*_6_) δ: 1.37 (s, 9H, Me_3_C); 1.33–1.43 (m, 4H, H-2, H-3); 2.42 (t, 2H, *J* = 6.4 Hz, CH_2_NH, H-4); 2.88–2.90 (m, 2H, CH_2_NHCO, H-1); 3.53 (s, 2H, ArCH_2_NH); 4.20 (s, 4H, H-2ʹ, H-3ʹ, Ar); 6.75–6.80 (br s, 1H, NHCO); 6.75–6.80 (m, 3H, H-5ʹ, H-7ʹ, H-8ʹ, Ar). ^13^C-NMR (DMSO-*d*_6_) δ: 26.8 (C-2); 27.4 (C-3); 28.2 (OC(CH_3_)_3_); 39.8 (C-4); 48.2 (C-1); 52.3 (ArCH_2_NH); 63.9 (C-2ʹ, C-3ʹ, Ar); 64.0 (C-2ʹ, C-3ʹ, Ar); 77.2 (OC(CH_3_)_3_); 116.4 (C-7ʹ, Ar); 116.5 (C-8ʹ, Ar); 120.6 (C-5ʹ, Ar); 134.0 (C-6ʹ, Ar); 141.9 (C-4aʹ, C-8aʹ, Ar); 143.0 (C-4aʹ, C-8aʹ, Ar); 155.5 (C=O).

#### 3.1.4. Standard Procedure for the Preparation of Hydrochloride Salts **6a**–**f** after Deprotection of *N*-Boc Monoprotected Diamines **5a**–**f**

In a 100 mL two-necked round-bottomed flask provided with a magnetic stirrer and condenser, *N-*Boc monoprotected diamine **5** (12.1 mmol, 1 equiv.) was solubilized in 1,4-dioxane (80 mL) at room temperature under vigorous stirring for 10 min. To this homogeneous solution was added dropwise for 1 h a solution of 6 M HCl (20 mL). The reaction mixture was stirred during 4 h at 25 °C and was concentrated in a rotary evaporator under reduced pressure for elimination of volatile compounds. To the crystallized crude reaction mixture was added 60 mL of anhydrous Et_2_O and after triturating, the insoluble salt **6** was collected by filtration, on a Büchner funnel (porosity N°4) then washed with 6 × 10 mL of Et_2_O. The desired salt **6** was dried under high vacuum (10^−2^ Torr) at 25 °C for 1 h that gave a yellowish or white powder and was further used without purification.

*N-1-Phenylmethyl-butane-1,4-diamine hydrochloride* (**6a**). Compound **6a** was prepared in 84% yield (2.18 g) from (4-phenylmethylamino-butyl)-carbamic acid *tert-*butyl ester **5a** (3.37 g, 12.1 mmol) according to the standard procedure as yellowish powder. Mp > 260 °C. ^1^H-NMR (DMSO-*d*_6_) δ: 1.57–1.67 (m, 2H, CH_2_, H-3); 1.70–1.81 (m, 2H, CH_2_, H-2); 2.79 (t, 2H, *J =* 7.3 Hz, CH_2_NH, H-4); 2.89 (t, 2H, *J =* 7.5 Hz, CH_2_NH_2_, H-1); 4.10 (s, 2H, CH_2_NH, H-4); 7.4–7.45 (m, 3H, H-3ʹ, H-4ʹ, H-5ʹ, Ar); 7.58–7.62 (m, 2H, H-2ʹ, H-6ʹ, Ar); 8.19 (br s, 2H, NH_2_); 9.57 (br s, 1H, NH). ^13^C-NMR (DMSO-*d*_6_) δ: 22.3 (C-3); 24.0 (C-2); 38.0 (C-4); 45.5 (C-1); 49.7 (ArCH_2_NH); 128.5 (C-3ʹ, C-5ʹ, Ar); 128.8 (C-4ʹ, Ar); 130.1 (C-2ʹ, C-6ʹ, Ar); 132.0 (C-1ʹ, Ar).

*N-1-(4-Methoxy-phenylmethyl)-butane1,4-diamine hydrochloride* (**6b**). Compound **6b** was prepared in 97% yield (2.87 g) from (4-methoxyphenylmethylamino-butyl)-carbamic acid *tert-*butyl ester **5b** (3.73 g, 12.1 mmol) according to the standard procedure as pale brownish powder. Mp > 260 °C. ^1^H-NMR (DMSO-*d*_6_) δ: 1.57–1.66 (m, 2H, H-3); 1.69–1.79 (m, 2H, H-2); 2.77–2.84 (m, 4H, H-1, H-4); 3.76 (s, 3H, OCH_3_); 4.03 (s, 2H, ArCH_2_NH); 6.97 (d, 2H, *J* = 8.7 Hz, H-2ʹ, H-6ʹ, Ar); 7.51 (d, 2H, *J* = 8.7 Hz, H-3ʹ, H-5ʹ, Ar), 8.15 (br s, 2H, NH_2_); 9.41 (br s, 1H, NH). ^13^C-NMR (DMSO-*d*_6_) δ: 22.3 (C-3); 24.0 (C-2); 38.0 (C-4); 45.3 (C-1); 49.2 (ArCH_2_NH); 55.2 (OCH_3_); 113.9 (C-2ʹ, C-6ʹ, Ar); 123.7 (C-1ʹ, Ar); 131.6 (C-3ʹ, C-5ʹ, Ar); 159.6 (C-4ʹ, Ar).

*N-1-(2-Chloro-phenylmethyl)-butane1,4-diamine hydrochloride* (**6c**). Compound **6c** was prepared in 98% yield (2.95 g) from (2-chlorophenylmethylamino-butyl)-carbamic acid *tert-*butyl ester **5c** (3.79 g, 12.1 mmol) according to the standard procedure as pale brownish powder. Mp = 206–208 °C. ^1^H-NMR (DMSO-*d*_6_) δ: 1.60–1.69 (m, 2H, H-3); 1.74–1.84 (m, 2H, H-2); 2.73–2.84 (m, 2H, CH_2_NH, H-4); 2.93–3.02 (m, 2H, CH_2_NH_2_, H-1); 4.23 (t, 2H, *J =* 5.9 Hz, ArCH_2_NH); 7.39–7.48 (m, 2H, H-4ʹ, H-5ʹ, Ar); 7.52–7.56 (m, 1H, H-6ʹ, Ar); 7.81–7.85 (m, 1H, H-3ʹ, Ar); 8.18 (br s, 2H, NH_2_, 2H); 9.68 (br s, 1H, NH). ^13^C-NMR (DMSO-*d*_6_) δ: 22.3 (C-3); 24.0 (C-2); 38.0 (C-4); 46.1 (C-1); 46.7 (ArCH_2_NH); 127.5 (C-5ʹ, Ar); 129.5 (C-4ʹ, Ar); 129.9 (C-1ʹ, Ar); 130.7 (C-6ʹ, Ar); 131.9 (C-3ʹ, Ar); 133.5 (C-2ʹ, Ar).

*N-1-(3-Methoxy-phenylmethyl)-butane-1,4-diamine hydrochloride* (**6d**). Compound **6d** was prepared in 98% yield (2.98 g) from [4-(3-methoxy-phenylmethylamino)-butyl]-carbamic acid tert-butyl ester **5d** (3.73 g, 12.1 mmol) according to the standard procedure as pale brownish powder. Mp = 190–192 °C. _1_H-NMR (DMSO-*d*_6_) δ: 1.58–1.67 (m, 2H, H-3); 1.71–1.81 (m, 2H, H-2); 2.78 (t, 2H, *J* = 7.3 Hz, CH_2_NH, H-4); 2.87 (t, 2H, *J* = 7.6 Hz, CH_2_NH_2_, H-1); 3.77 (s, 3H, OCH_3_); 4.08 (t, 2H, *J =* 5.6 Hz, ArCH_2_NH); 6.94–6.97 (m, 1H, H-5ʹ, Ar); 7.12–7.14 (m, 1H, H-6ʹ, Ar); 7.29–7.35 (m, 2H, H-2ʹ, H-4ʹ, Ar); 8.18 (br s, 2H, NH_2_); 9.58 (br s, 1H, NH). ^13^C-NMR (DMSO-*d*_6_) δ: 22.3 (C-3); 24.0 (C-2); 38.0 (C-4); 45.5 (C-1); 49.7 (ArCH_2_NH); 55.2 (OCH_3_); 114.5 (C-5ʹ, Ar); 115.5 (C-6ʹ, Ar); 122.0 (C-4ʹ, Ar); 129.6 (C-2ʹ, Ar); 133.4 (C-1ʹ, Ar); 159.2 (C-3ʹ, Ar).

*N-1-Benzo[1,3]dioxol-5-ylmethyl-butane-1,4-diamine hydrochloride* (**6e**). Compound **6e** was prepared in 72% yield (2.25 g) from {4-[(benzo[1,3]dioxol-5-ylmethyl)-amino]-butyl}-carbamic acid *tert-*butyl ester **5e** (3.90 g, 12.1 mmol) according to the standard procedure as pale brownish powder. Mp > 260 °C. ^1^H-NMR (DMSO-*d*_6_) δ: 1.58–1.67 (m, 2H, H-3); 1.71–1.81 (m, 2H, H-2); 2.78 (t, 2H, *J* = 7.3 Hz, CH_2_NH, H-4); 2.87 (t, 2H, *J* = 7.6 Hz, CH_2_NH_2_, H-1); 3.77 (s, 3H, OCH_3_); 4.08 (t, 2H, *J =* 5.6 Hz, ArCH_2_NH); 6.94–6.97 (m, 1H, H-5ʹ, Ar); 7.12–7.14 (m, 1H, H-6ʹ, Ar); 7.29–7.35 (m, 2H, H-2ʹ, H-4ʹ, Ar); 8.18 (br s, 2H, NH_2_); 9.58 (br s, 1H, NH). ^13^C-NMR (DMSO-*d*_6_) δ: 22.3 (C-3); 24.0 (C-2); 38.0 (C-4); 45.2 (C-1); 49.4 (ArCH_2_NH); 101.2 (C-2ʹ, Ar); 108.2 (C-6ʹ, Ar); 110.4 (C-7ʹ, Ar); 124.1 (C-4ʹ, Ar); 125.4 (C-5ʹ, Ar); 147.2 (C-3aʹ, C-7aʹ, Ar); 147.6 (C-3aʹ, C-7aʹ, Ar).

*N-1-(2,3-Dihydro-benzo[1,4]dioxin-6-ylmethyl)-butane-1,4-diamine hydrochloride* (**6f**). Compound **6e** was prepared in 92% yield (3.04 g) from {4-[(2,3-dihydro-benzo[1,4]dioxin-6-ylmethyl)-amino]-butyl}-carbamic acid *tert-*butyl ester **5f** (4.07 g, 12.1 mmol) according to the standard procedure as pale brownish powder. Mp = 239–241 °C. ^1^H-NMR (DMSO-*d*_6_) δ: 1.57–1.66 (m, 2H, H-3); 1.68–1.78 (m, 2H, H-2); 2.73–2.82 (m, 4H, H-1, H-4); 3.97 (t, 2H, *J =* 5.4 Hz, ArCH_2_NH); 4.24 (s, 4H, OCH_2_CH_2_O); 6.87 (d, 1H, *J =* 8.2 Hz, H-7ʹ, Ar); 7.01–7.04 (m, 1H, H-8ʹ, Ar); 7.15 (m, 1H, H-5ʹ, Ar); 8.18 (br s, 2H, NH_2_); 9.43 (br s, 1H, NH). ^13^C-NMR (DMSO-*d*_6_) δ: 22.3 (C-3); 24.0 (C-2); 38.0 (C-4); 45.2 (C-1); 49.1 (ArCH_2_NH); 64.0 (C-2ʹ, Ar); 64.1 (C-3ʹ, Ar); 117.0 (C-7ʹ, Ar); 119.0 (C-8ʹ, Ar); 123.2 (C-5ʹ, Ar); 124.7 (C-6ʹ, Ar); 143.1 (C-4aʹ, C-8aʹ, Ar); 143.8 (C-4aʹ, C-8aʹ, Ar).

#### 3.1.5. Standard Procedure for the Preparation of *N*-Substituted 5-Arylidene Rhodanine Derivatives **9a**–**f** under Microwave Irradiation Using “One-Pot Two-steps” Protocol

In a 10 mL glass tube were placed successively bis-(carboxymethyl)trithiocarbonate **7** (0.11 g, 0.50 mol, 1 equiv.), dimethoxyethane (2 mL), triethylamine (135 mL, 101 mg, 1 mmol, 2 equiv. or 202 mL, 152 mg, 1.5 mmol, 3 equiv.) and hydrochloride salt **6** (0.5 mmol, 1 equiv.). The glass tube was sealed with a snap cap and placed in the Monowave^®^ 300 Anton Paar microwave cavity (*p* = 800 Watt). The reaction mixture was irradiated at 90 °C for 15–60 min. under vigorous magnetic stirring. After microwave dielectric heating, the crude reaction mixture was allowed to cool down at room temperature, aldehyde **3** (0.5 mol, 1 equiv.) was added to the cooled reaction mixture which was immediately submitted to microwave irradiation at 110 °C for 5–30 min. After cooling down to room temperature, the volatile compounds of the reaction mixture were removed in a rotary evaporator under reduced pressure. To the crude reaction mixture was added 4 mL of MeOH and after triturating, the insoluble product **9** was collected by filtration on a Büchner funnel (porosity N°4), washed with deionized water (5 mL), triturated and mixing with ethanol (3 × 5 mL) during 3 h, washed successively with ethanol (5 mL) and anhydrous ether (5 × 2 mL), then dried under high vacuum (10^−2^ Torr) at 25 °C for 1 h. The desired compound **9** was eventually purified by recrystallization in EtOH after control by ^1^H-NMR in solution of CDCl_3_/TFA (98:2).

*(5Z)3-[4-(4-Methoxyphenylmethylamino)butyl]-5-(1,3-benzodioxol-5-ylmethylene)-2-thioxo-1,3-thiazolidin-4-one* (**9a**). According to the standard procedure, compound **9a** was prepared in 10% yield (23 mg) from *N-1*-(4-methoxy-phenylmethyl)-butane-1,4-diamine hydrochloride **6b** (122.3 mg, 0.5 mmol, 1 equiv.), triethylamine (202 μL, 152 mg, 1.5 mmol, 3 equiv.) and piperonaldehyde **3e** (75.1 mg, 0.5 mmol, 1 equiv.) after 30 min. at 90 °C, followed by a second reaction time of 30 min. at 110 °C (for condensation step with **3e**), which gave **9a** as a yellowish powder. Mp = 170–229 °C (decomposition). ^1^H-NMR (CDCl_3_/TFA 98:2) δ: 1.61–1.69 (m, 4H, CH_2_, H-2ʹʹ, H-3ʹʹ); 3.05–3.11 (m, 2H, ArCH_2_NH); 3.73 (s, 3H, OCH_3_); 4.00–4.08 (m, 4H, CH_2_, H-1ʹʹ, H-4ʹʹ), 5.97 (s, 2H, CH_2_, H-2ʹ); 6.80–7.16 (m, 7H, H-2ʹʹʹ, H-3ʹʹʹ, H-5ʹʹʹ, H-6ʹʹʹ, H-4ʹ, H-6ʹ, H-7ʹ, Ar); 7.55 (br s, 1H, NH); 7.61 (s, 1H, HC=C). ^13^C-NMR (CDCl_3_/TFA 98:2) δ: 23.1 (C-2ʹʹ, 3ʹʹ); 23.8 (C-3ʹʹ, 2ʹʹ); 44.2 (C-4ʹʹ, C-1ʹʹ); 47.0 (ArCH_2_NH); 52.1 (C-1ʹʹ, C-4ʹʹ); 55.5 (OCH_3_); 102.2 (C-2ʹ); 109.4 (C-2ʹʹʹ, C-6ʹʹʹ); 109.5 (C-3ʹʹʹ, C-5ʹʹʹ); 115.0 (C-6ʹ); 120.0 (C-1ʹʹʹ); 127.5 (C=CH); 128.2 (C-7ʹ); 131.2 (C-4ʹ); 135.0 (C=CH); 148.8 (C-5ʹ); 148.9 (C-4ʹʹʹ); 150.6 (C-3ʹa); 150.9 (C-7ʹa); 169.4 (C=O, C-4); 192.9 (C=S, C-2). HRMS, *m*/*z*: 457.1256 found (calculated for C_23_H_25_N_2_O_4_S_2_ [M + H]^+^ requires 457.1253).

*(5Z)3-[4-(4-Methoxyphenylmethylamino)butyl]-5-(4-methoxybenzylidene)-2-thioxo-1,3-thiazolidin-4-one* (**9b**). According to the standard procedure, compound **9b** was prepared in 6% yield (13.3 mg) from *N-1*-(4-methoxy-phenylmethyl)-butane-1,4-diamine hydrochloride **6b** (122.3 mg, 0.5 mmol, 1 equiv.), triethylamine (202 μL, 152 mg, 1.5 mmol, 3 equiv.) and 4-methoxybenzaldehyde **3b** (68.1 mg, 0.5 mmol, 1 equiv.) after 30 min. at 90 °C, followed by a second reaction time of 30 min. at 110 °C (for condensation step with **3b**), which gave **9b** as a yellowish powder. Mp = 253–255 °C. ^1^H-NMR (CDCl_3_/TFA 98:2) δ: 1.67–1.73 (m, 4H, CH_2_, H-2ʹʹ, H-3ʹʹ); 3.07–3.13 (m, 2H, ArCH_2_NH); 3.81 (s, 6H, ArOCH_3_); 4.04–4.11 (s, 4H, CH_2_, H-1ʹʹ, H-4ʹʹ), 6.94 (dd, 4H, *J =* 8.8 Hz, H-2ʹ, H-6ʹ, H-2ʹʹʹ, H-6ʹʹʹ, Ar); 7.40 (d, 4H, *J =* 8.8 Hz, H-3ʹ, H-5ʹ, H-3ʹʹʹ, H-5ʹʹʹ, Ar); 7.60 (br s, 1H, NH); 7.67 (s, 1H, CH=). ^13^C-NMR (CDCl_3_/TFA 98:2) δ: 24.3 (C-2ʹʹ, C-3ʹʹ); 44.0 (C-1ʹʹ, C-4ʹʹ); 46.8 (ArCH_2_NH); 55.6 (C-4ʹ, OCH_3_, C-4ʹʹʹ, OCH_3_); 115.1 (C-2ʹ, C-6ʹ, C-2ʹʹʹ, C-6ʹʹʹ); 119.6 (C-1ʹ, C-1ʹʹʹ); 126.0 (C=); 133.1 (C-3ʹ, C-5ʹ, C-3ʹʹʹ, C-5ʹʹʹ); 130.5 (C-5ʹ, C-5ʹʹʹ); 134.6 (CH=); 162.0 (C-4ʹ, C-4ʹʹʹ); 169.0 (C=O, C-4); 193.2 (C=S, C-2). HRMS, *m*/*z*: 443.1463 found (calculated for C_23_H_27_N_2_O_3_S_2_ [M + H]^+^ requires 443.1460).

*(5Z)3-[4-(4-Methoxyphenylmethylamino)butyl]-5-(2,3-dihydro-1,4-benzodioxin-6-ylmethylene)-2-thioxo-1,3-thiazolidin-4-one* (**9c**). According to the standard procedure, compound **9c** was prepared in 5% yield (11.8 mg) from *N-1*-(4-methoxy-phenylmethyl)-butane-1,4-diamine hydrochloride **6b** (122.3 mg, 0.5 mmol, 1 equiv.), triethylamine (202 μL, 152 mg, 1.5 mmol, 3 equiv.) and 2,3-dihydro-1,4-benzodioxin-6-carboxaldehyde **3f** (82.1 mg, 0.5 mmol, 1 equiv.) after 30 min. at 90 °C, followed by a second reaction time of 30 min. at 110 °C (for condensation step with **3f**), which gave **9c** as a yellowish powder. Mp = 255–261 °C. ^1^H-NMR (CDCl_3_/TFA 98:2) δ: 1.68 (s, 4H, CH_2_, H-2ʹʹ, H-3ʹʹ); 3.03–3.08 (m, 2H, ArCH_2_NH); 3.73 (s, 3H, OCH_3_); 4.07 (s, 4H, CH_2_, H-1ʹʹ, H-4ʹʹ), 4.20–4.25 (m, 2H, CH_2_, H-2ʹ, H-3ʹ); 6.84–6.97 (m, 7H, H-5ʹ, H-7ʹ, H-8ʹ, H-2ʹʹʹ, H-3ʹʹʹ, H-5ʹʹʹ, H-6ʹʹʹ, Ar); 7.58 (s, 1H, CH=); 7.60 (br s, 1H, NH). ^13^C-NMR (CDCl_3_/TFA 98:2) δ: 24.2 (C-2ʹʹ, C-3ʹʹ); 44.1 (C-4ʹʹ, C-1ʹʹ); 47.1 (ArCH_2_NH); 55.0 (OCH_3_); 64.2 (C-2ʹ); 64.8 (C-3ʹ); 118.4 (C-5ʹ, C-7ʹ, C-8ʹ); 119.7 (C-2ʹʹʹ, C-6ʹʹʹ); 120.3 (C=); 125.7 (C-3ʹʹʹ, C-5ʹʹʹ); 126.8 (C-6ʹ); 135.0 (CH=); 143.9 (C-8ʹa); 146.7 (C-4ʹa); 169.5 (C=O, C-4); 193.2 (C=S, C-2). HRMS, *m*/*z*: 471.1412 found (calculated for C_24_H_27_N_2_O_4_S_2_ [M + H]^+^ requires 471.1415).

*(5Z)3-[4-(4-Methoxyphenylmethylamino)butyl]-5-(4-hydroxy-3-methoxybenzylidene)-2-thioxo-1,3-thiazolidin-4-one* (**9d**). According to the standard procedure, compound **9d** was prepared in 30% yield (68.8 mg) from *N-1*-(4-methoxy-phenylmethyl)-butane-1,4-diamine hydrochloride **6b** (122.3 mg, 0.5 mmol, 1 equiv.), triethylamine (202 μL, 152 mg, 1.5 mmol, 3 equiv.) and 4-hydroxy-3-methoxybenzaldehyde **3g** (76.1 mg, 0.5 mmol, 1 equiv.) after 30 min. at 90 °C, followed by a second reaction time of 30 min. at 110 °C (for condensation step with **3g**), which gave **9d** as a yellowish powder. Mp = 191–193 °C. ^1^H-NMR (CDCl_3_/TFA 98:2) δ: 1.69 (m, 4H, CH_2_, H-2ʹʹ, H-3ʹʹ); 3.06–3.07 (m, 2H, ArCH_2_NH); 3.72 (s, 3H, C-4ʹʹʹ, OCH_3_); 3.86 (s, 3H, C-3ʹ, OCH_3_); 4.04–4.08 (m, 4H, CH_2_, H-1ʹʹ, H-4ʹʹ), 6.81–7.17 (m, 7H, H-2ʹ, H-5ʹ, H-6ʹ, H-2ʹʹʹ, H-3ʹʹʹ, H-5ʹʹʹ, H-6ʹʹʹ, Ar); 7.55 (br s, 1H, NH); 7.60 (s, 1H, CH=C). ^13^C-NMR (CDCl_3_/TFA 98:2) δ: 23.1 (C-2ʹʹ, C-3ʹʹ); 23.9 (C-2ʹʹ, C-3ʹʹ); 43.1 (C-1ʹʹ, C-4ʹʹ); 47.0 (ArCH_2_NH); 55.5 (ArOCH_3_); 56.1 (ArOCH_3_); 112.6 (C-6ʹ); 115.0 (C-2ʹʹʹ, C-6ʹʹʹ); 115.6 (C-2ʹ); 118.9 (C-1ʹʹʹ); 121.2 (C=); 125.9 (C-1ʹ); 126.8 (C-5ʹ); 131.2 (C-3ʹʹʹ, C-5ʹʹʹ); 135.9 (CH=); 147.2 (C-4ʹʹʹ); 148.9 (C-3ʹ); 160.8 (C-4ʹ); 169.4 (C=O, C-4); 193.1 (C=S, C-2). HRMS, *m*/*z*: 459.1412 found (calculated for C_23_H_27_N_2_O_4_S_2_ [M + H]^+^ requires 459.1416).

*(5Z)3-[4-(3-Methoxyphenylmethylamino)butyl]-5-(1,3-benzodioxol-5-ylmethylene)-2-thioxo-1,3-thiazolidin-4-one* (**9e**). According to the standard procedure, compound **9e** was prepared in 21% yield (48.0 mg) from *N-1*-(3-methoxy-phenylmethyl)-butane-1,4-diamine hydrochloride **6d** (122.3 mg, 0.5 mmol, 1 equiv.), triethylamine (202 μL, 152 mg, 1.5 mmol, 3 equiv.) and piperonaldehyde **3e** (75.1 mg, 0.5 mmol, 1 equiv.) after 15 min. at 90 °C, followed by a second reaction time of 15 min. at 110 °C (for condensation step with **3e**), which gave **9e** as a yellowish powder. Mp = 178–246 °C (decomposition). ^1^H-NMR (CDCl_3_/TFA 98:2) δ: 1.82 (s, 4H, CH_2_, H-2ʹʹ, H-3ʹʹ); 3.19–3.22 (m, 2H, ArCH_2_NH); 3.83 (s, 3H, OCH_3_); 4.13-4.23 (m, 4H, CH_2_, H-1ʹʹ, H-4ʹʹ), 6.10 (s, 2H, CH_2_, H-2ʹ); 6.92–7.28 (m, 6H, H-2ʹʹʹ, H-4ʹʹʹ, H-6ʹʹʹ, H-4ʹ, H-6ʹ, H-7ʹ, Ar), 7.33–7.38 (m, 1H, H-5ʹʹʹ, Ar), 7.55 (br s, 1H, NH); 7.70 (s, 1H, CH=). ^13^C-NMR (CDCl_3_/TFA 98:2) δ: 23.1 (C-2ʹʹ, C-3ʹʹ); 23.9 (C-3ʹʹ, C-2ʹʹ); 42.9 (C-4ʹʹ, C-1ʹʹ); 46.8 (ArCH_2_NH); 52.1 (C-1ʹʹ, C-4ʹʹ); 55.3 (OCH_3_); 102.1 (C-2ʹ); 109.3 (C-6ʹʹʹ); 109.4 (C-5ʹʹʹ); 114.9 (C-4ʹʹʹ); 115.9 (C-2ʹʹʹ); 119.8 (C-1ʹʹʹ); 121.8 (C-6ʹ); 127.4 (C=); 127.6 (C-5ʹ); 127.9 (C-7ʹ); 130.7 (C-4ʹ); 134.5 (CH=); 148.8 (C-7ʹa); 150.5 (C-3ʹa); 160.3 (C-3ʹʹʹ); 168.5 (C=O, C-4); 193.1 (C=S, C-2). HRMS, *m*/*z*: 457.1259 found (calculated for C_23_H_25_N_2_O_4_S_2_ [M + H]^+^ requires 457.1257).

*(5Z)3-[4-(3-Methoxyphenylmethylamino)butyl]-5-(2,3-dihydro-1,4-benzodioxin-6-ylmethylene)-2-thioxo-1,3-thiazolidin-4-one* (**9f**). According to the standard procedure, compound **9c** was prepared in 9% yield (21.2 mg) from *N-1*-(3-methoxy-phenylmethyl)-butane-1,4-diamine hydrochloride **6d** (122.3 mg, 0.5 mmol, 1 equiv.), triethylamine (202 μL, 152 mg, 1.5 mmol, 3 equiv.) and 2,3-dihydro-1,4-benzodioxin-6-carboxaldehyde **3f** (82.1 mg, 0.5 mmol, 1 equiv.) after 15 min. at 90 °C, followed by a second reaction time of 15 min. at 110 °C (for condensation step with **3f**), which gave **9f** as a yellowish powder. Mp = 247–251 °C. ^1^H-NMR (CDCl_3_/TFA 98:2) δ: 1.84 (s, 4H, CH_2_, H-2ʹʹ, H-3ʹʹ); 3.04–3.12 (m, 2H, ArCH_2_NH); 3.95 (s, 3H, OCH_3_); 4.23 (s, 4H, CH_2_, H-1ʹʹ, H-4ʹʹ); 4.37–4.40 (m, 4H, H-2ʹ, H-3ʹ); 6.99–7.28 (m, 6H, H-2ʹʹʹ, H-4ʹʹʹ, H-5ʹʹʹ, H-6ʹʹʹ, H-5ʹ, H-7ʹ, H-8ʹ, Ar); 7.60 (br s, 1H, NH); 7.74 (s, 1H, CH=). ^13^C-NMR (CDCl_3_/TFA 98:2) δ: 24.1 (C-2ʹʹ, C-3ʹʹ); 44.1 (C-1ʹʹ, C-4ʹʹ); 47.3 (ArCH_2_NH); 64.2 (C-2ʹ); 64.8 (C-3ʹ); 118.5 (C-5ʹ, C-7ʹ, C-8ʹ); 119.8 (C-4ʹʹʹ, C-6ʹʹʹ); 125.9 (C-2ʹʹʹ, C-5ʹʹʹ); 126.8 (C=); 135.3 (CH=); 143.8 (C-8ʹa); 146.6 (C-4ʹa); 168.5 (C=O, C-4); 193.3 (C=S, C-2). HRMS, *m*/*z*: 471.1412 found (calculated for C_24_H_27_N_2_O_4_S_2_ [M + H]^+^ requires 471.1415).

*(5Z)3-[4-(3-Methoxyphenylmethylamino)butyl]-5-(3-methoxybenzylidene)-2-thioxo-1,3-thiazolidin-4-one* (**9g**). According to the standard procedure, compound **9g** was prepared in 5% yield (11.1 mg) from *N-1*-(3-methoxy-phenylmethyl)-butane-1,4-diamine hydrochloride **6d** (122.3 mg, 0.5 mmol, 1 equiv.), triethylamine (202 μL, 152 mg, 1.5 mmol, 3 equiv.) and 3-methoxybenzaldehyde **3d** (68.1 mg, 0.5 mmol, 1 equiv.) after 30 min. at 90 °C, followed by a second reaction time of 30 min at 110 °C (for condensation step with **3d**), which gave **9g** as a yellowish powder. Mp = 225–227 °C. ^1^H-NMR (CDCl_3_/TFA 98:2) δ: 1.73 (s, 4H, CH_2_, H-2ʹʹ, H-3ʹʹ); 3.06–3.10 (m, 2H, ArCH_2_NH); 3.80 (s, 6H, ArOCH_3_); 4.11 (s, 4H, CH_2_, H-1ʹʹ, H-4ʹʹ), 6.93–6.96 (m, 4H, H-2ʹ, H-6ʹ, H-2ʹʹʹ, H-6ʹʹʹ, Ar); 7.04 (d, 2H, *J =* 7.7 Hz, H-4ʹ, H-4ʹʹʹ, Ar), 7.32 (m, 2H, H-5ʹ, H-5ʹʹʹ, Ar), 7.55 (br s, 1H, NH); 7.66 (s, 1H, CH=). ^13^C-NMR (CDCl_3_/TFA 98:2) δ: 24.3 (C-2ʹʹ, C-3ʹʹ); 44.0 (C-1ʹʹ, C-4ʹʹ); 47.1 (ArCH_2_NH); 55.5 (ArOCH_3_); 115.3 (C-6ʹ, C-6ʹʹʹ); 117.3 (C-2ʹ, C-2ʹʹʹ); 123.1 (C=); 123.6 (C-4ʹ, C-4ʹʹʹ); 130.5 (C-5ʹ, C-5ʹʹʹ); 134.0 (CH=); 134.5 (C-1ʹ, C-1ʹʹʹ); 160.0 (C-3ʹ, C-3ʹʹʹ); 168.5 (C=O, C-4); 193.3 (C=S, C-2). HRMS, *m*/*z*: 443.1463 found (calculated for C_23_H_27_N_2_O_3_S_2_ [M + H]^+^ requires 443.1465).

*(5Z)3-[4-(1,3-Benzodioxol-5-ylmethylamino)butyl]-5-(1,3-benzodioxol-5-ylmethylene)-2-thioxo-1,3-thiazolidin-4-one* (**9h**). According to the standard procedure, compound **9h** was prepared in 59% yield (139 mg) from *N-1-* benzo[1,3]dioxol-5-ylmethyl-butane-1,4-diamine hydrochloride **6e** (129.4 mg, 0.5 mmol, 1 equiv.), triethylamine (135 μL, 101 mg., 1 mmol, 2 equiv.) and piperonaldehyde **3e** (75.1 mg, 0.5 mmol, 1 equiv.) after 15 min. at 90 °C, followed by a second reaction time of 15 min. at 110 °C (for condensation step with **3e**), which gave **9h** as a yellowish powder. Mp = 245–256 °C. ^1^H-NMR (CDCl_3_/TFA 98:2) δ: 1.74 (s, 4H, CH_2_, H-2ʹʹ, H-3ʹʹ); 3.04–3.05 (m, 2H, ArCH_2_NH); 4.05–4.07 (m, 4H, CH_2_, H-1ʹʹ, H-4ʹʹ); 5.91 (s, 2H, CH_2_, H-2ʹʹʹ); 6.00 (s, 2H, CH_2_, H-2ʹ); 6.72–7.01 (m, 6H, H-4ʹ, H-6ʹ, H-7ʹ, H-4ʹʹʹ, H-6ʹʹʹ, H-7ʹʹʹ, Ar); 7.58 (s, 1H, CH=); 7.65 (br s, 1H, NH);. ^13^C-NMR (CDCl_3_/TFA 98:2) δ: 23.1 (C-2ʹʹ, C-3ʹʹ); 24.0 (C-3ʹʹ); 43.1 (C-1ʹʹ, C-4ʹʹ); 46.5 (ArCH_2_NH); 51.9 (C-4ʹʹ, C-1ʹʹ); 101.8 (C-2ʹ, C-2ʹʹʹ); 102.2 (C-2ʹʹʹ, C-2ʹ); 109.0 (C-6ʹʹʹ); 109.3 (C-6ʹ); 109.4 (C-7ʹʹʹ); 109.8 (C-7ʹ); 119.8 (C-5ʹʹʹ); 122.6 (C=); 124.3 (C-4ʹʹʹ); 127.4 (C-4ʹ); 128.1 (C-5ʹ); 134.9 (CH=); 148.6 (C-7ʹa); 148.9 (C-3ʹa); 149.2 (C-7ʹʹʹa); 150.6 (C-3ʹʹʹa); 168.9 (C=O, C-4); 193.0 (C=S, C-2). HRMS, *m*/*z*: 471.1048 found (calculated for C_23_H_23_N_2_O_5_S_2_ [M + H]^+^ requires 471.1049).

*(5Z)3-[4-(1,3-Benzodioxol-5-ylmethylamino)butyl]-5-(2,3-dihydro-1,4-benzodioxin-6-ylmethylene)-2-thioxo-1,3-thiazolidin-4-one* (**9i**). According to the standard procedure, compound **9i** was prepared in 59% yield (143 mg) from *N-1-*benzo[1,3]dioxol-5-ylmethyl-butane-1,4-diamine hydrochloride **6e** (129.4 mg, 0.5 mmol, 1 equiv.), triethylamine (135 μL, 101 mg., 1 mmol, 2 equiv.) and 2,3-dihydro-1,4-benzodioxin-6-carboxaldehyde **3f** (82.1 mg, 0.5 mmol, 1 equiv.) after 15 min. at 90 °C, followed by a second reaction time of 15 min. at 110 °C (for condensation step with **3f**), which gave **9i** as a yellowish powder. Mp = 253–257 °C. ^1^H-NMR (CDCl_3_/TFA 98:2) δ: 1.68 (s, 4H, CH_2_, H-2ʹʹ, H-3ʹʹ); 3.05–3.06 (m, 2H, ArCH_2_NH); 4.03–4.04 (m, 4H, CH_2_, H-1ʹʹ, H-4ʹʹ); 4.20–4.25 (m, 4H, CH_2_, H-2ʹ, H-3ʹ); 5.89 (s, 2H, CH_2_, H-2ʹʹʹ); 6.70 (s, 3H, H-4ʹʹʹ, H-6ʹʹʹ, H-7ʹʹʹ, Ar); 6.85–6.96 (m, 3H, H-5ʹ, H-7ʹ, H-8ʹ, Ar); 7.50 (br s, 1H, NH); 7.55 (s, 1H, CH=). ^13^C-NMR (CDCl_3_/TFA 98:2) δ: 23.1 (C-2ʹʹ, C-3ʹʹ); 23.8 (C-3ʹʹ, C-2ʹʹ); 43.1 (C-1ʹʹ, C-4ʹʹ); 47.0 (ArCH_2_NH); 52.4 (C4ʹʹ, C-1ʹʹ); 64.2 (C-2ʹ, C-3ʹ); 64.8 (C-3ʹ, C-2ʹ); 101.8 (C-2ʹʹʹ); 109.1 (C-6ʹʹʹ); 109.5 (C-7ʹʹʹ); 118.5 (C-7ʹ); 119.7 (C-5ʹʹʹ); 119.8 (C-8ʹ); 122.3 (C=); 124.1 (C-4ʹʹʹ); 125.9 (C-5ʹ); 126.6 (C-6ʹ); 135.5 (CH=); 144.0 (C-7ʹʹʹa); 146.9 (C-3ʹʹʹa); 148.6 (C-8ʹa); 149.4 (C-4ʹa); 169.5 (C=O, C-4); 193.2 (C=S, C-2). HRMS, *m*/*z*: 485.1205 found (calculated for C_24_H_25_N_2_O_5_S_2_ [M + H]^+^ requires 485.1203).

*(5Z)3-[4-(1,3-Benzodioxol-5-ylmethylamino)butyl]-5-(4-hydroxy-benzylidene)-2-thioxo-1,3-thiazolidin-4-one* (**9j**). According to the standard procedure, compound **9j** was prepared in 34% yield (75.2 mg) from *N-1-*benzo[1,3]dioxol-5-ylmethyl-butane-1,4-diamine hydrochloride **6e** (129.4 mg, 0.5 mmol, 1 equiv.), triethylamine (135 μL, 101 mg, 1 mmol, 2 equiv.) and 4-hydroxybenzaldehyde **3h** (61.1 mg, 0.5 mmol, 1 equiv.) after 15 min. at 90 °C, followed by a second reaction time of 15 min. at 110 °C (for condensation step with **3h**), which gave **9i** as a orange powder. Mp = 93–124 °C (decomposition). ^1^H-NMR (CDCl_3_/TFA 98:2) δ: 1.59–1.73 (m, 4H, CH_2_, H-2ʹʹ, H-3ʹʹ); 3.01–3.13 (m, 2H, ArCH_2_NH); 4.00–4.05 (m, 4H, CH_2_, H-1ʹʹ, H-4ʹʹ); 5.88 (s, 2H, CH_2_, H-2ʹʹʹ); 6.66–6.69 (m, H-4ʹʹʹ, H-6ʹʹʹ, H-7ʹʹʹ, Ar); 6.86 (dd, 2H, *J* = 8.7 Hz, H-2ʹ, H-6ʹ, Ar); 7.32 (dd, 2H, *J* = 8.7 Hz, H-3ʹ, H-5ʹ, Ar); 7.55 (br s, 1H, NH); 7.60 (s, 1H, CH=, 1H). ^13^C-NMR (CDCl_3_/TFA 98:2) δ: 22.5 (C-2ʹʹ, C-3ʹʹ); 23.2 (C-3ʹʹ, C-2ʹʹ); 42.5 (C-1ʹʹ, C-4ʹʹ); 46.4 (ArCH_2_NH); 51.9 (C-4ʹʹ, C-1ʹʹ); 101.2 (C-2ʹʹʹ); 108.5 (C-6ʹʹʹ); 108.8 (C-7ʹʹʹ); 116.1 (C-2ʹ, C-6ʹ); 118.6 (C-5ʹʹʹ); 121.7 (C=); 123.4 (C-4ʹʹʹ); 125.6 (C-1ʹ); 132.9 (C-3ʹ, C-5ʹ); 134.8 (CH=); 148.0 (C-7ʹʹʹa); 148.8 (C-3ʹʹʹa); 157.6 (C-4ʹ); 169.0 (C=O, C-4); 192.6 (C=S, C-2). HRMS, *m*/*z*: 443.1099 found (calculated for C_22_H_23_N_2_O_4_S_2_ [M + H]^+^ requires 443.1095).

*(5Z)3-[4-(1,3-Benzodioxol-5-ylmethylamino)butyl]-5-(4-hydroxy-3-methoxybenzylidene)-2-thioxo-1,3-thiazolidin-4-one* (**9k**). According to the standard procedure, compound **9k** was prepared in 46% yield (109 mg) from *N-1-*benzo[1,3]dioxol-5-ylmethyl-butane-1,4-diamine hydrochloride **6e** (129.4 mg, 0.5 mmol, 1 equiv.), triethylamine (202 μL, 152 mg, 1.5 mmol, 3 equiv.) and 4-hydroxy-3-methoxybenzaldehyde **3g** (76.1 mg, 0.5 mmol, 1 equiv.) after 30 min. at 90 °C, followed by a second reaction time of 30 min. at 110 °C (for condensation step with **3g**), which gave **9k** as a red powder. Mp = 200–203 °C. ^1^H-NMR (CDCl_3_/TFA 98:2) δ: 1.71 (s, 4H, CH_2_, H-2ʹʹ, H-3ʹʹ); 3.05–3.06 (m, 2H, ArCH_2_NH); 3.88 (s, 3H, OCH_3_); 4.04 (s, 4H, CH_2_, H-1ʹʹ, H-4ʹʹ); 5.91 (s, 2H, CH_2_, H-2ʹʹʹ); 6.74 (s, 3H, H-4ʹʹʹ, H-6ʹʹʹ, H-7ʹʹʹ, Ar); 6.87–7.02 (m, 3H, H-2ʹ, H-5ʹ, H-6ʹ, Ar); 7.58 (s, 1H, CH=); 7.65 (br s, 1H, NH). ^13^C-NMR (CDCl_3_/TFA 98:2) δ: 23.1 (C-2ʹʹ, C-3ʹʹ); 23.9 (C-3ʹʹ, C-2ʹʹ); 42.9 (C-1ʹʹ, C-4ʹʹ); 46.6 (ArCH_2_NH); 52.1 (C-4ʹʹ, C-1ʹʹ); 56.1 (OCH_3_); 101.8 (C-2ʹʹʹ); 109.0 (C-6ʹʹʹ); 109.6 (C-7ʹʹʹ); 112.3 (C-6ʹ); 115.5 (C-2ʹ); 119.1 (C-5ʹʹʹ); 122.7 (C=); 124.0 (C-5ʹ); 125.8 (C-1ʹ); 126.5 (C-4ʹʹʹ); 134.9 (CH=); 147.1 (C-3ʹ); 148.6 (C-7ʹʹʹa); 148.8 (C-4ʹ); 149.2 (C-3ʹʹʹa); 168.5 (C=O, C-4); 193.2 (C=S, C-2). HRMS, *m*/*z*: 473.1205 found (calculated for C_23_H_25_N_2_O_5_S_2_ [M + H]^+^ requires 473.1206).

*(5Z)-[4-(2,3-Dihydro-benzo[1,4]dioxin-6-ylmethylamino)butyl]-5-(1,3-benzodioxol-5-ylmethylene)-2-thioxo-1,3-thiazolidin-4-one* (**9l**). According to the standard procedure, compound **9l** was prepared in 15% yield (36.3 mg) from *N-1-*(2,3-dihydro-benzo[1,4]dioxin-6-ylmethyl)-butane-1,4-diamine hydrochloride **6f** (136.4 mg, 0.5 mmol, 1 equiv.), triethylamine (202 μL, 152 mg, 1.5 mmol, 3 equiv.) and piperonaldehyde **3e** (75.1 mg, 0.5 mmol, 1 equiv.) after 15 min. at 90 °C, followed by a second reaction time of 15 min. at 110 °C (for condensation step with **3e**), which gave **9l** as a yellowish powder. Mp = 172–233 °C (decomposition). ^1^H-NMR (CDCl_3_/TFA 98:2) δ: 1.66–1.69 (m, 4H, CH_2_, H-2ʹʹ, H-3ʹʹ); 3.03–3.10 (m, 2H, ArCH_2_NH); 4.03–4.07 (m, 4H, CH_2_, H-1ʹʹ, H-4ʹʹ); 4.16–4.19 (m, 4H, CH_2_, H-2ʹʹʹ, H-3ʹʹʹ); 5.97 (s, 2H, CH_2_, H-2ʹ); 6.66–7.00 (m, 6H, H-4ʹ, H-6ʹ, H-7ʹ, H-5ʹʹʹ, H-7ʹʹʹ, H-8ʹʹʹ, Ar); 7.55 (br s, 1H, NH); 7.59 (s, 1H, CH=). ^13^C-NMR (CDCl_3_/TFA 98:2) δ: 23.8 (C-2ʹʹ, C-3ʹʹ); 24.2 (C-3ʹʹ); 43.1 (C-1ʹʹ, C-4ʹʹ); 47.0 (ArCH_2_NH); 52.1 (C-4ʹʹ, C-1ʹʹ); 64.2 (C-2ʹʹʹ); 64.4 (C-3ʹʹʹ); 102.2 (C-2ʹ); 109.4 (C-7ʹʹʹ); 109.5 (C-8ʹʹʹ); 118.5 (C-5ʹʹʹ); 119.5 (C-6ʹʹʹ); 122.0 (C=); 122.9 (C-6ʹ); 127.3 (C-5ʹ); 128.2 (C-7ʹ); 135.0 (C-4ʹ); 135.5 (CH=); 143.9 (C-4ʹʹʹa); 145.1 (C-8ʹʹʹa); 150.6 (C-3ʹa); 150.8 (C-7ʹa); 168.5 (C=O, C-4); 193.0 (C=S, C-2). HRMS, *m*/*z*: 485.1205 found (calculated for C_24_H_25_N_2_O_5_S_2_ [M + H]^+^ requires 485.1206).

*(5Z)3-[4-(2,3-Dihydro-benzo[1,4]dioxin-6-ylmethylamino)butyl]-5-(2,3-dihydro-benzo*[1,4]*dioxin-6-ylmethylene)-2-thioxo-1,3-thiazolidin-4-one* (**9m**). According to the standard procedure, compound **9m** was prepared in 7% yield (17.5 mg) from *N-1-*(2,3-dihydro-benzo[1,4]dioxin-6-ylmethyl)-butane-1,4-diamine hydrochloride **6f** (136.4 mg, 0.5 mmol, 1 equiv.), triethylamine (202 μL, 152 mg, 1.5 mmol, 3 equiv.) and 2,3-dihydro-1,4-benzodioxin-6-carboxaldehyde **3f** (82.1 mg, 0.5 mmol, 1 equiv.) after 15 min. at 90 °C, followed by a second reaction time of 15 min. at 110 °C (for condensation step with **3f**), which gave **9m** as a yellowish powder. Mp = 170–243 °C (decomposition). ^1^H-NMR (CDCl_3_/TFA 98:2) δ: 1.64–1.69 (m, 4H, CH_2_, H-2ʹʹ, H-3ʹʹ); 3.04–3.10 (m, 2H, ArCH_2_NH); 4.03–4.08 (m, 4H, CH_2_, H-1ʹʹ, H-4ʹʹ); 4.16–4.23 (m, 8H, CH_2_, H-2ʹ, H-3ʹ, H-2ʹʹʹ, H-3ʹʹʹ); 6.75–7.57 (m, 6H, H-5ʹ, H-7ʹ, H-8ʹ, H-5ʹʹʹ, H-7ʹʹʹ, H-8ʹʹʹ, Ar); 7.60 (br s, 1H, NH); 8.77 (s, 1H, CH=). ^13^C-NMR (CDCl_3_/TFA 98:2) δ: 24.2 (C-2ʹʹ, C-3ʹʹ); 44.0 (C-1ʹʹ, C-4ʹʹ); 64.2 (C-2ʹ, C-2ʹʹʹ); 64.8 (C-3ʹ, C-3ʹʹʹ); 118.4 (C-7ʹ, C-7ʹʹʹ); 119.6 (C-8ʹ, C-8ʹʹʹ); 120.3 (C=); 125.6 (C-5ʹ, C-5ʹʹʹ); 126.8 (C-6ʹ, C-6ʹʹʹ); 134.5 (CH=); 144.0 (C-8ʹa, C-8ʹʹʹa); 146.6 (C-4ʹa, C-4ʹʹʹa); 166.6 (C=O, C-4); 182.5 (C=S, C-2). HRMS, *m*/*z*: 499.1364 found (calculated for C_25_H_27_N_2_O_5_S_2_ [M + H]^+^ requires 499.1366).

*(5Z)3-[4-(2,3-Dihydro-benzo[1,4]dioxin-6-ylmethylamino)butyl]-5-(4-hydroxy-3-methoxybenzylidene)-2-thioxo-1,3-thiazolidin-4-one* (**9n**). According to the standard procedure, compound **9n** was prepared in 34% yield (82.7 mg) from *N-1-*(2,3-dihydro-benzo[1,4]dioxin-6-ylmethyl)-butane-1,4-diamine hydrochloride **6f** (136.4 mg, 0.5 mmol, 1 equiv.), triethylamine (202 μL, 152 mg, 1.5 mmol, 3 equiv.) and 4-hydroxy-3-methoxybenzaldehyde **3g** (76.1 mg, 0.5 mmol, 1 equiv.) after 30 min. at 90 °C, followed by a second reaction time of 30 min. at 110 °C (for condensation step with **3g**), which gave **9n** as a red powder. Mp = 202–204 °C. ^1^H-NMR (CDCl_3_/TFA 98:2) δ: 1.69 (s, 4H, CH_2_, H-2ʹʹ, H-3ʹʹ); 3.06 (s, 2H, ArCH_2_NH); 3.86 (s, 3H, OCH_3_); 4.03 (s, 4H, CH_2_, H-1ʹʹ, H-4ʹʹ); 4.16 (s, 4H, CH_2_, H-2ʹʹʹ, H-3ʹʹʹ); 6.67–7.03 (m, 6H, H-2ʹ, H-5ʹ, H-6ʹ, H-5ʹʹʹ, H-7ʹʹʹ, H-8ʹʹʹ, Ar); 7.60 (s, 1H, CH=). ^13^C-NMR (CDCl_3_/TFA 98:2) δ: 23.1 (C-2ʹʹ or 3ʹʹ); 23.8 (C-3ʹʹ ou C-2ʹʹ); 43.1 (C-1ʹʹ ou 4ʹʹ); 47.0 (ArCH_2_NH); 52.1 (C-4ʹʹ, C-1ʹʹ); 56.1 (OCH_3_); 64.4 (C-2ʹʹʹ, C-3ʹʹʹ); 64.4 (C-3ʹʹʹ, C-2ʹʹʹ); 112.6 (C-6ʹ); 115.6 (C-2ʹ); 118.4 (C-7ʹʹʹ); 118.6 (C-8ʹʹʹ); 119.0 (C-6ʹʹʹ); 122.0 (C=); 122.9 (C-5ʹʹʹ); 125.9 (C-1ʹ); 126.8 (C-5ʹ); 135.9 (CH=); 143.9 (C-8ʹʹʹa); 145.1 (C-3ʹa); 147.2 (C-4ʹʹʹa); 148.9 (C-4ʹ);169.4 (C=O, C-4); 193.1 (C=S, C-2). HRMS, *m*/*z*: 487.1361 found (calculated for C_24_H_27_N_2_O_5_S_2_ [M + H]^+^ requires 487.1359).

### 3.2. Biochemistry Section

#### 3.2.1. Protein Kinase Assay Buffers

*Buffer A*: 10 mM MgCl_2_, 1 mM EGTA, 1 mM DTT, 25 mM Tris-HCl pH 7.5, 50 µg heparin/mL.

*Buffer B:* 60 mM β-glycerophosphate, 15 mM *p-*nitrophenyl-phosphate, 25 mM Mops (pH 7.2), 5 mM EGTA, 15 mM MgCl_2_, 1 mM DTT, 1 mM sodium vanadate, 1 mM phenylphosphate.

#### 3.2.2. Kinase Preparations and Assays

Kinase activities for each enzyme were assayed in buffer A (25 mM Tris-HCl pH 7.5, 10 mM MgCl_2_, 1 mM EGTA, 1 mM DTT, 50 µg/mL Heparin, BSA 0.15 mg/mL) or B (60 mM β-glycerophosphate, 30 mM *p*-Nitrophenylphosphate, 25 mM MOPS, 5 mM EGTA, 15 mM MgCl_2_, 1 mM DTT, 0.1 mM Na vanadate), with their corresponding substrates, in the presence of 15 µM [γ-^33^P] ATP (3000 Ci/mmol; 10 mCi/mL) in a final volume of 30 µL. After 30 min incubation at 30 °C, the reaction was stopped by harvesting, using a FilterMate harvester (Packard, Meriden, CT, USA), onto P81 phosphocellulose papers (GE Healthcare, Velizy-Villacoublay, France) which were washed in 1% phosphoric acid. Scintillation fluid was added and the radioactivity measured in a Packard counter. Blank values were subtracted and activities calculated as pmoles of phosphate incorporated during the 30 min incubation. The activities were expressed in a percentage of the maximal activity, *i.e.*, in the absence of inhibitors. Controls were performed with appropriate dilutions of DMSO. *CK1* and *Haspin* peptide substrates were obtained from Proteogenix (Oberhausbergen, France).

*CDK5/p25* (human, recombinant) was prepared as previously described [31]. Its kinase activity was assayed in buffer B, with 1 mg histone H1/mL.

*Casein kinase 1 (CK1δ/ε)* (porcine brain, native) was assayed with 0.67 µg of CKS peptide (RRKHAAIGpSAYSITA) [32].

*GSK-3*α/β (porcine brain, native) was assayed, as described for CDK5/p25 but in Buffer A and using a GSK-3 specific substrate (GS-1: YRRAAVPPSPSLSRHSSPHQSpEDEEE) (pS stands for phosphorylated serine) [33]. GS-1 was synthesized by Millegen (Labege, France).

*Haspin* kinase domain (*HsHaspin*-kd aa 470 to 798) encoding cDNA, obtain by RT-PCR, was cloned into pGex-6P-3. The fusion protein was expressed in *Escherichia coli* strain BL21-KRX (Promega, Madison, WI, USA) and purified by affinity chromatography on glutathione-agarose beads (Sigma). Haspin-kd activity was assayed with 3 µM Histone H3 (1-21) peptide, a specific Haspin substrate, (ARTKQTARKSTGGKAPRKQLA), in Buffer H (MOPS 25 mM pH 7.5; 10 mM MgCl_2_).

### 3.3. Cell Culture and Survival Assays

Skin diploid fibroblastic cells were provided by BIOPREDIC International Company (Rennes, France). Caco2 (Ref ECACC: 86010202), Huh-7D12 (Ref ECACC: 01042712), MDA-MB-231 (Ref ECACC: 92020424), HCT-116 (Ref ECACC: 91091005), PC3 (Ref ECACC: 90112714), NCI-H727 (Ref ECACC: 94060303) cell lines were obtained from the ECACC collection and HaCaT (from Cell Lines Service, Eppelheim, Germany). Cells were grown according to ECACC recommendations [34]. The toxicity test of the compounds on these cells was as follows: 2 × 10^3^ cells for HCT-116 cells or 4 × 10^3^ for the other cells were seeded in 96 multiwell plates in triplicate and left for 24 h for attachment, spreading and growing. Then, cells were exposed for 48 h to increasing concentrations of the compounds, ranging from 0.1 to 25 μM in a final volume of 120 μL of culture medium. Cells were fixed in cooled solution of 90% ethanol/5% acetic acid, nuclei were stained with Hoechst 3342 (Sigma) and counted using automated imaging analysis (Cellomics Arrayscan VTI/HCS Reader, Thermo/Scientific, Waltham, MA, USA). The IC_50_ were graphically determined.

## 4. Conclusions

In summary, we have developed, in this preliminary project, a new route to (5*Z*) 3-(4-arylmethylamino)butyl-5-arylidene-2-thioxo-1,3-thiazolidine-4-ones **9**. Starting from commercial butane-1,4-diamine, the process involved six steps and the key step is a solution phase “one-pot two-steps” approach for the construction of the 2-thioxo-1,3-thiazolidine-4-one platform under microwave dielectric heating followed by Knoevenagel condensation for installation of the 5-arylidene moiety as second point of diversity. This methodology offered the possibility of preparing a library of fourteen new compounds in moderate to good yields and, the targeted compounds **9a**–**n** have been built with a *Z*-geometry. The *in vitro* inhibition of cell proliferation was carried out on a panel of seven representative tumoral cell lines and the compounds **9a**–**n** were also evaluated against four protein kinases. Among all of these compounds, the compound **9j** turned out to be interesting because it presented selective micromolar inhibition activity on *Ss*CK1 (IC_50_ 1.4 μM). Molecules **9h** and **9i** were also bioactive on *Ss*CK1 and *Hs*CDK5-p25. The current results are the starting point of a new larger program within our group to investigate intensively the biological properties of these new inhibitors with potential application in Alzheimer’s disease or in cancer.
